# Adipocyte-secreted BMP8b mediates adrenergic-induced remodeling of the neuro-vascular network in adipose tissue

**DOI:** 10.1038/s41467-018-07453-x

**Published:** 2018-11-26

**Authors:** Vanessa Pellegrinelli, Vivian J. Peirce, Laura Howard, Samuel Virtue, Dénes Türei, Martina Senzacqua, Andrea Frontini, Jeffrey W. Dalley, Antony R. Horton, Guillaume Bidault, Ilenia Severi, Andrew Whittle, Kamal Rahmouni, Julio Saez-Rodriguez, Saverio Cinti, Alun M. Davies, Antonio Vidal-Puig

**Affiliations:** 1 Metabolic Research Laboratories, Institute of Metabolic Science, Addenbrooke’s Hospital, University of Cambridge, Cambridge, CB2 0QQ UK; 2 School of Biosciences, Cardiff University, Museum Avenue, Cardiff, CF10 3AT UK; 3 European Molecular Biology Laboratory (EMBL), Structural and Computational Biology Unit, Meyerhofstrasse 1, D-69117 Heidelberg, Germany; 4 European Molecular Biology Laboratory (EMBL), European Bioinformatics Institute (EBI), Cambridge, CB10 1SD UK; 5 RWTH Aachen University, Faculty of Medicine, Joint Research Centre for Computational Biomedicine, MTI2 Wendlingweg 2, D-52074 Aachen, Germany; 6 Department of Experimental and Clinical Medicine, Center of Obesity, Università Politecnica delle Marche, 60126 Ancona, Italy; 7 Department of Public Health, Experimental and Forensic Medicine, University of Pavia, 27100 Pavia, Italy; 8 Department of Psychology, University of Cambridge, Downing Street, Cambridge, CB2 3EB UK; 9 Department of Psychiatry, University of Cambridge, Addenbrooke’s Hospital, Cambridge, CB2 0QQ UK; 10 Department of Pharmacology, University of Iowa, Iowa City, IA 52242 USA; 11 Wellcome Trust Sanger Institute, Wellcome Trust Genome Campus, Hinxton, Cambridge, CB10 1SA UK

## Abstract

Activation of brown adipose tissue-mediated thermogenesis is a strategy for tackling obesity and promoting metabolic health. BMP8b is secreted by brown/beige adipocytes and enhances energy dissipation. Here we show that adipocyte-secreted BMP8b contributes to adrenergic-induced remodeling of the neuro-vascular network in adipose tissue (AT). Overexpression of *bmp8b* in AT enhances browning of the subcutaneous depot and maximal thermogenic capacity. Moreover, BMP8b-induced browning, increased sympathetic innervation and vascularization of AT were maintained at 28 °C, a condition of low adrenergic output. This reinforces the local trophic effect of BMP8b. Innervation and vascular remodeling effects required BMP8b signaling through the adipocytes to 1) secrete neuregulin-4 (NRG4), which promotes sympathetic axon growth and branching in vitro, and 2) induce a pro-angiogenic transcriptional and secretory profile that promotes vascular sprouting. Thus, BMP8b and NRG4 can be considered as interconnected regulators of neuro-vascular remodeling in AT and are potential therapeutic targets in obesity.

## Abstract (web summary)

Enhancing thermogenesis is a promising therapeutic strategy for promoting metabolic health. Here the authors show that adipocyte-secreted BMP8b contributes to optimizing the thermogenic response by remodeling of the neuro-vascular networks in brown and white adipose tissue.

## Introduction

Cold-induced activation of brown adipose tissue (BAT) enables energy dissipation in the form of heat through uncoupling of fatty acid oxidation from adenosine triphosphate production^1^. In humans, thermogenically active BAT (e.g., para-aortic and cervical^2,3^) is detectable in lean individuals, indicating that promoting BAT-induced thermogenesis in obese humans may represent a safe therapeutic strategy to improve their metabolic health. Beige cells, another specialized cell type present in white adipose tissue (WAT), fundamentally share similar thermogenic mechanisms but are developmentally and functionally distinct from classical BAT cells^4^. These differences between classic brown and beige cells open the opportunity for alternative therapeutic opportunities to increase their thermogenic capacity by specific activators.

BAT activity/expansion and beige cell recruitment are regulated by cold-mediated activation of the sympathetic nervous system (SNS) and its chemical messenger norepinephrine (NE)^5^. In addition, optimal activation and maintenance of BAT and beige adipocytes require the coordinated expansion of ancillary sympathetic nervous and vascular networks. Synchronization between sympathetic tone input, thermogenic demands and adipose tissue (AT) remodeling is likely to involve the fine-tuned interplay of other AT cellular components (i.e., immune, endothelial and adipocyte cells), whose interaction and activity should not be assumed to be directly dependent on the SNS activity^6,7^. Additionally, there is increasing evidence that BAT also fulfils a secretory role releasing factors such as fibroblast growth factor 21 (FGF21), neuregulin-4 (NRG4), bone morphogenetic proteins (BMPs) and angiogenic factors^8^ that contribute to autocrine, paracrine and endocrine functions and may be required for intercellular coordination and tissue remodeling.

Several studies have highlighted the importance of vascular remodeling in activation/maintenance of browning and BAT thermogenic response. Beige cell recruitment in WAT is associated with increased vascularization^6,9^ and, indeed, beige and brown adipocytes might be derived from vascular precursors^10,11^. Additionally, pro-angiogenic factor overexpression, e.g., vascular endothelial cell growth factor (VEGF), increases vascular density and expression of thermogenic genes in both BAT and WAT. Conversely, neutralization of VEGF reduces BAT activity coupled to decreased vascular density, highlighting the interdependence of matched thermogenesis and angiogenesis^12–14^. The mechanisms driving cold-induced remodeling of the neural network are not fully understood. NRG4 is a novel “batokine” that may contribute to the adipocyte-neuronal cross-talk by promoting neurite outgrowth^15^. This may complement the suggested effects of NRG4 controlling de novo liver lipid synthesis in liver and preventing diet-induced insulin resistance and hepatic steatosis^16^. Taken together, this supports the importance of the neuro-vascular network regulating thermogenesis, and also the requirement that sophisticated mechanisms exist to spatially and temporally titrate both the expansion of the vascular network and sympathetic innervation to adjust SNS input to meet thermogenic demands^10,11^.

BMP8b is the only BMP predominantly expressed in mature brown adipocytes and uniquely sensitizes brown adipocytes to adrenergic input, amplifying their thermogenic response^17,18^. Its physiological relevance is attested by the reduced thermogenic response and impairment in diet- and cold-induced thermogenesis observed in *Bmp8b*^–/–^ mice^17^.

In our study, we focus on the BMP8b peripheral effects to understand how BMP8b potentiates the adrenergically mediated thermogenic response. We used transgenic mice in which *bmp8b* is overexpressed in AT in combination with cellular models to identify a cell-autonomous peripheral role of BMP8b that optimizes the thermogenic response by promoting remodeling of the neuro-vascular networks in BAT and WAT.

## Results

### *Bmp8b* overexpression enhances AT browning and thermogenesis

We generated a transgenic mouse in which expression of the murine *Bmp8b* gene is under control of the adipocyte-specific apolipoprotein 2 (*Fabp4*) promoter^19,20^ (*Bmp8b* transgenic (TG) mice). *Bmp8b* gene expression in *Bmp8b* TG mice was enhanced in BAT, subcutaneous (ScW) and gonadal WAT (GnW) with no induced or negligible expression in the liver and hypothalamus (Supplementary figure 1a, b). No major compensatory change of other AT-related members of the BMP family were observed (Supplementary Figure 1c). *Bmp8b* overexpression in AT was expected to inform about the role of increased *bmp*8b levels in response to cold exposure (1 week, 5 °C) in ScW (Supplementary figure 1d).

**Fig. 1:**
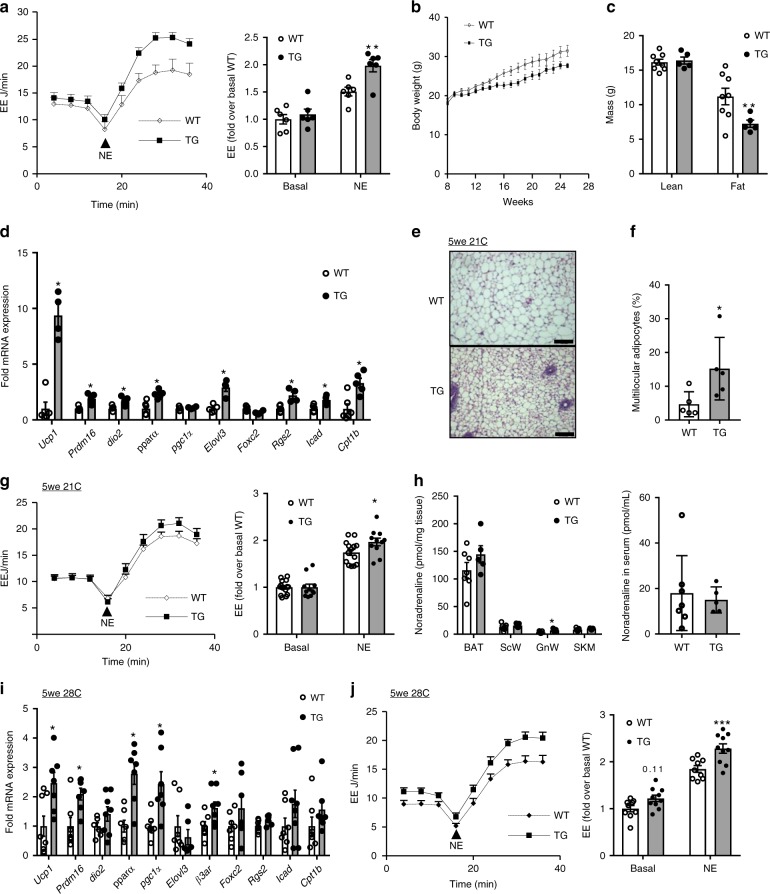
AT BMP8b overexpression enhances browning and maximal thermogenic capacity. **a** NE-stimulated EE in 12-week-old WT and *Bmp8b* TG mice housed at 21 °C, expressed as EE over time (genotype effect *p* = 0.018; NE effect *p* = 0.00001) and fold increase over basal condition (B), *n* = 6. **b**, **c** Body weights (**b**) and body composition (**c**) from WT and TG female mice aged 8–25 weeks and fed a 45% HFD; *n* = 5–8. **d** mRNA levels of thermogenic genes, genes revealing or involved in adrenergic sensitivity and signaling and genes involved in fatty acid oxidation in ScW 4-week-old WT and *Bmp8b* TG mice fed chow diet and kept at 21 °C; *n* = 4–6. **e** Representative images of hematoxylin and eosin (H&E) staining of ScW from 5-week-old WT and *Bmp8b* TG female mice and kept at 21 °C. Scale bar, 100 µm. **f** Quantification of the percentage of total adipocyte population showing multilocularity in ScW from 5-week-old WT and *Bmp8b* TG female mice; *n* = 5. **g** NE-stimulated EE in 5-week-old WT and TG mice housed at 21 °C, expressed as EE over time (genotype effect *p* = 0.025; NE effect 9.7 E–11) and fold increase over basal condition, *n* = 11–14. **h** Noradrenaline measurement in adipose tissue and serum from 12-week-old *bmp8b* WT and TG animals; *n* = 5–7. **i** mRNA levels of thermogenic genes, genes revealing or involved in adrenergic sensitivity and signaling, genes involved in fatty acid oxidation in ScW from 4-week-old WT and *Bmp8b* TG female mice born and housed at 28 °C, *n* = 9. **j** NE-stimulated EE in 5-week-old WT and *Bmp8b* TG mice born and housed at 28 °C, expressed as EE over time (genotype effect *p* = 0.002; NE effect 12.1 E–11) and fold increase over basal condition, *n* = 9–10. Mean ± s.e.m. are presented. *P < 0.05, **p < 0.01, ***p<0.001, repeated measures ANOVA with Sidak’s post-hoc multiple comparisons test (**a**, **c**, **j**); *p < 0.05, compared to WT using *t*-test (**f**, **h**); *p < 0.05, compared to WT using multiple *t*-test with FDR determined using the two-stage linear step-up procedure of Benjamini, Krieger and Yekutieli (**d**, **i**)

Adult *Bmp8b* TG mice exhibited increased energy expenditure (EE) in response to NE injection compared to wild-type (WT) littermates, consistent with greater thermogenic capacity (Fig. 1a). This was a cell-autonomous effect as ScW adipocytes from adult TG mice also displayed an increased β-adrenergic-stimulated lipolysis response compared to WTs (Supplementary figure 2a). WT and *Bmp8b* TG mice fed chow diet and housed at 21 °C did not show body weight and food intake differences (Supplementary figure 2b, c). However, after 5 months of high-fat diet (HFD), the *Bmp8b* TG mice tended to be leaner than the WTs (Fig. 1b, c). At that stage, we did not observe a significant decrease in basal glycemia or improved insulin sensitivity and basal EE remained similar (Supplementary figure 2d-f).

Characterization of ScW thermogenic gene expression from 20-week-old chow-fed *Bmp8b* TG mice only showed increased *Pgc1α* gene expression (Supplementary figure 2g) compared to WTs. However, gene expression analysis in post-weaning 5-week-old *Bmp8b* TG mice revealed a broad up-regulation in ScW of thermogenic genes (*Ucp-1* and *Pdrm16*) and genes related to fatty acid oxidation (*Cpt1b*) (Fig. 1d). This pro-thermogenic molecular phenotype in ScW contrasted with unaltered expression of thermogenic genes in BAT and GnW from both post-weaning and adult *Bmp8b* TG mice (Supplementary figure 2h-j). UCP1 protein levels were detectable in ScW of most *Bmp8b* TG mice in basal conditions compared to WT 5-week-old animals (Supplementary figure 2k), whereas UCP1 protein levels in BAT from *Bmp8b* TG BAT were similar to WT mice. Accordingly, ScW from Bmp8b TG showed decreased adipocyte area (Supplementary figure 2l) and greater percentage of multilocular adipocytes compared to WTs (Fig. 1e, f). As in adult mice, post-weaning *Bmp8b* TG mice were similar in body weight, body composition and basal EE to WTs (Supplementary Fig. 2m-o). However, when adrenergically stimulated, mice had increased maximal thermogenic capacity compared to WTs, as indicated by the greater EE of *Bmp8b* TG mice following NE injection (Fig. 1g).

Central injection of BMP8b has been shown to induce browning in the ScW through activation of the hypothalamic ventromedial nucleus. Consequently, we investigated if over-activated SNS in *bmp8b* TG mice was responsible for the browning and thermogenic phenotype. Baseline serum free fatty acid levels were similar in WTs and Bmp8b TGs (Supplementary Figure 3a). Despite a slight increase in the GnW, no differences in noradrenaline levels between 12-week-old *bmp8b* WT and TG mice were observed neither in the serum nor skeletal muscle and other fat depots, in particular the ScW, in which the thermogenic activation and neuro-vascular remodeling was the most potent (Fig. 1h). Finally, no significant differences were observed in basal sympathetic tone to epididymal WAT or BAT, or in BAT sympathetic nerve activation evoked by cooling (Supplementary Figure 3b, c). However, to assess the effects of BMP8b in a state where sympathetic tone is minimized, adult *Bmp8b* TG mice were switched to 28 °C prior being given HFD. Under these conditions, *Bmp8b* TG mice lost the leaner phenotype (decreased fat mass) observed when given a HFD at ambient temperature, suggesting that these effects might be NE mediated (Supplementary Figure 3d, e). However, despite the low adrenergic output, *bmp8b* overexpression was sufficient to maintain the beige cell recruitment and up-regulation of thermogenic genes *ucp1*, *prdm16*, *dio2*, *pparα* and *pgc1α* in the ScW compared to WT mice. No differences were observed in BAT and GnW (Fig. 1i, Supplementary Figure 3f-i). Likewise, despite being raised at 28 °C and lacking adrenergic priming, both basal and NE-stimulated EE were still significantly higher in post-weaning and adult *Bmp8b* TG mice compared to the WTs (Fig. 1j, Supplementary Figure 3j). Overall, our results indicate that the effects of BMP8b might not simply reflect increased SNS tone, but an enhanced thermogenesis resulting from a greater functionality of beige fat (i.e., beige cells and supportive networks).

### BMP8b modulates BAT and ScW sympathetic innervation in vivo

**Fig. 2:**
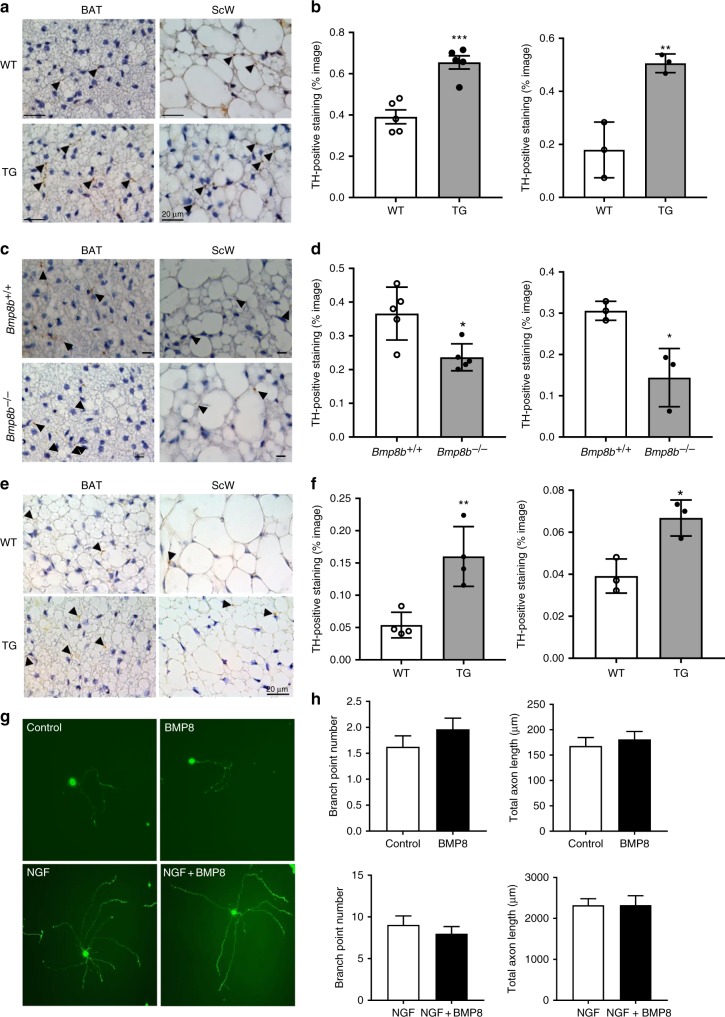
BMP8b overexpression increased innervation of the BAT and ScW. **a**–**d** Representative images of TH immunostaining (DAB) with nuclei in blue (hematoxylin) and quantification as a percentage of image, in ScW and BAT from 5-week-old *Bmp8b* TG versus WT (**a**, **b**) and *Bmp8b*^*+/+*^ versus *Bmp8b*^–/–^ (**c**, **d**) female mice; a black arrow in each image indicates a brown dot representing a parenchymal TH-positive fiber; *n* = 3–5. Scale bars, 20 µm (**a**) and 10 µm (**c**). **e**, **f** Representative images (**e**) of TH immunostaining (DAB) with nuclei in blue (hematoxylin) and quantification (**f**) as a percentage of image, in ScW and BAT from 5-week-old *Bmp8b* TG and WT female mice born and housed at 28 °C; a black arrow in each image indicates a brown dot representing a parenchymal TH-positive fiber. Scale bar, 20 µm; *n* = 5. **g**, **h** Representative images of primary sympathetic neurons cultured with caspase inhibitors (1 μg/ml) in the absence (control) or presence of NGF (0.1 ng/ml) and with or without BMP8 (80 ng/ml) (**g**). Data from a representative experiment with 50 neurons per condition: number of axon branch points per soma and total length of axon projections (**h**). Scale bar, 50 µm. Mean ± s.e.m. are presented; *p < 0.05, **p<0.01, ***p<0.001, compared to WT using a *t*-test (**b**, **d**, **f**)

As well as increasing sympathetic tone to BAT and ScW, chronic cold exposure also triggers sympathetic innervation^7^. Interestingly, some BMPs (e.g., 4 and 7) regulate nervous system development and promote in vitro dendritogenesis^21,22^. To determine whether BMP8b affected the level of AT sympathetic innervation in vivo, we stained for sympathetic fibers sections of BAT and ScW in *Bmp8b* TG, *Bmp8b*^*–/–*^ and WT littermate*s* (Fig. 2a, c). Quantification of tyrosine hydroxylase (TH)-positive staining^23,24^ in sections of 5-week-old mice revealed significantly increased nerve density in both BAT and ScW of *Bmp8b* TG mice compared with WT mice and significantly decreased nerve density in both BAT and ScW of *Bmp8b*^*–/–*^ mice compared with *Bmp8b*^*+/+*^ mice (Fig. 2b, d). Increased TH staining was observed in areas of white and beige adipocytes of *Bmp8b* TG mice while significant decreases were observed in both these areas of *Bmp8b*^–/–^ mice compared to WTs (Supplementary Figure 4a, b). Of relevance, BMP8b overexpression in mice born and housed at 28 °C still exhibited an increased innervation compared to WTs, supporting a plausible local neurotrophic role for BMP8b in the ScW (Fig. 2e, f).

Cold-induced increased sympathetic innervation involves nerve fiber branching and extension of preexisting neurons in BAT and ScW^7^. To determine if BMP8b influenced growth and branching of sympathetic axons we cultured primary sympathetic neurons (superior cervical ganglion (SCG)) with either BMP8b or nerve growth factor (NGF). Our finding that BMP8b did not enhance sympathetic axon growth in vitro, either in the presence or absence of NGF (Fig. 2g, h), raised the possibility that the effect of BMP8b on innervation was mediated by another factor.

### *Bmp8b* overexpression increased AT sympathetic innervation

**Fig. 3:**
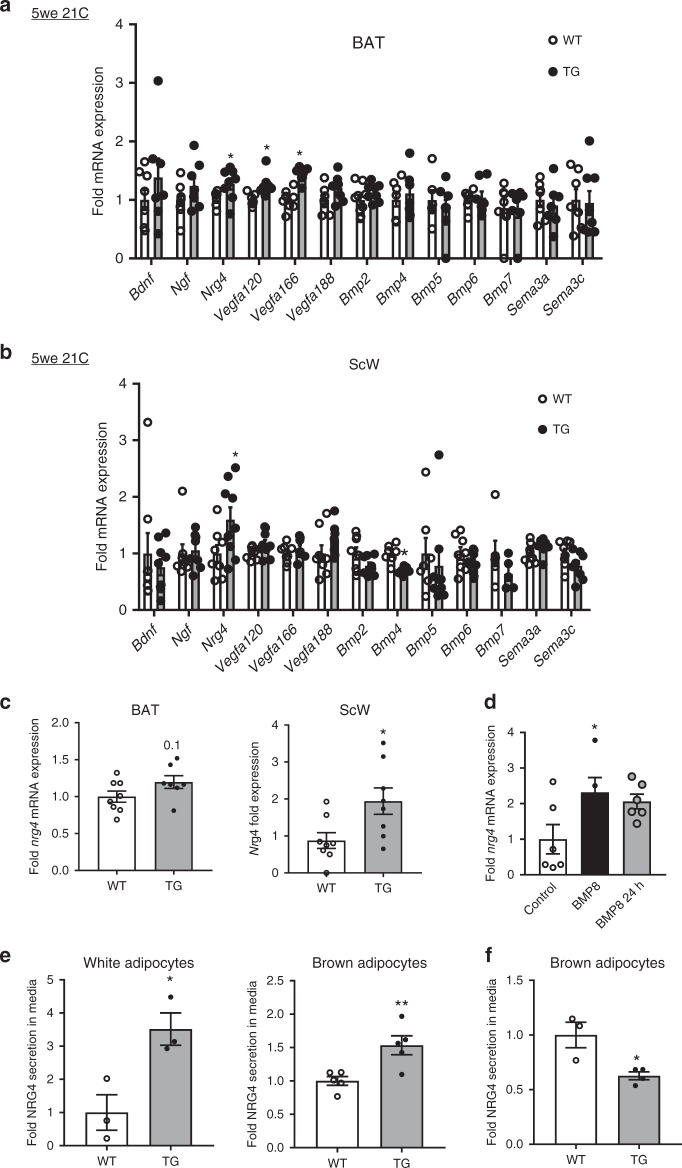
BMP8b-dependent regulation of NRG4 in AT and adipocytes. **a**, **b** Fold variation of mRNA levels of molecules that regulate sympathetic neuron survival, or axonal or dendritic guidance or growth in BAT (**a**) and ScW (**b**) from 5-week-old WT and *Bmp8b* TG female mice; *n* = 8–9. **c** NRG*4* mRNA levels in BAT and ScW from 4-week-old WT and TG female mice born and housed at 28 °C, *n* = 7–8. Data are represented normalized to the WT value; *p < 0.05 compared to WT using Student’s *t*-test (**e**–**h**). **d** Fold variation of NRG4 mRNA levels in differentiated brown adipocytes chronically (9 days, during differentiation) or acutely (24 h at day 8 of differentiation) treated with human recombinant BMP8 (red bar and pink bar, respectively). **e**–**g** ELISA analysis of NRG4 secretion of white unilocular (isolated from ScW) (**e**) and primary brown differentiated adipocytes (isolated from BAT) from 10-week-old *Bmp8b* TG versus WT (**e**, **f**) and *Bmp8b*^*+/+*^*versus Bmp8b*^–/–^ (**g**) mice; *n* = 3–5. Mean ± s.e.m. are presented; *p < 0.05, compared to WT using multiple *t*-test with FDR determined using the two-stage linear step-up procedure of Benjamini, Krieger and Yekutieli (**a**, **b**); *p < 0.05, **p<0.01 compared to WT using Mann–Whitney test (**c, e, f**); *p < 0.05, one-way ANOVA compared to control with Sidak’s post-hoc multiple comparisons test (**d**)

We then measured BAT and ScW from *Bmp8b* TG, *Bmp8b*^–/–^ and WT mice gene expression of sympathetic neurons growth regulators^16,21,25,26^. Among them, only *Nrg4* was significantly up-regulated in both BAT and ScW of *Bmp8b* TG mice (Fig. 3a, b) and down-regulated in the BAT of *Bmp8b*^*–/–*^ mice, compared to WT littermates. However, *Nrg4* remained unchanged in the GnW *from Bmp8b* TG mice (Supplementary Figure 4c-e). Since NRG4 levels in serum remained unchanged in *Bmp8b* TG and *Bmp8b*^–/–^ mice compared to their respective WTs, we posited a potential local action within AT (Supplementary Figure 4f, g). Of relevance, increased *Nrg4* expression in *Bmp8b* TG mice was maintained in the ScW of mice born and housed at 28 °C (Fig. 3c).

As previously described^15,16^, we verified that *Nrg4* was mainly expressed by the adipocyte fraction of BAT and ScW (Supplementary Figure 4h) and significantly induced in differentiated brown adipocytes following BMP8 treatment (Fig. 3d). This was a cell-autonomous effect since NRG4 was still over-produced by mature brown and white adipocytes isolated from AT of adult *Bmp8b* TG mice compared to adipocytes of WTs (Fig. 3e). Conversely, NRG4 secretion from mature brown adipocytes was decreased in adult *Bmp8b*^–/–^ mice (Fig. 3f).

The two murine protein-coding NRG4 isoforms require extracellular proteolytic cleavage to release the mature secreted form. Given that BACE1, AdAM10/17/19 have been shown to cleave NRG1^27,28^, we initially considered these matrix metalloproteinases as candidate proteases for NRG4 cleavage. We found that only *Adam10* messenger RNA (mRNA) was increased in both BMP8-treated and *Bmp8b* TG brown adipocytes compared to WTs (Supplementary Figure 4i, j). Pre-treatment of primary brown and white adipocytes with Galardin, a protease inhibitor, reduced NRG4 secretion into the media (Supplementary Figure 4k). These results support that BMP8b regulates NRG4 function at both transcriptional and post-transcriptional levels.

**Fig. 4:**
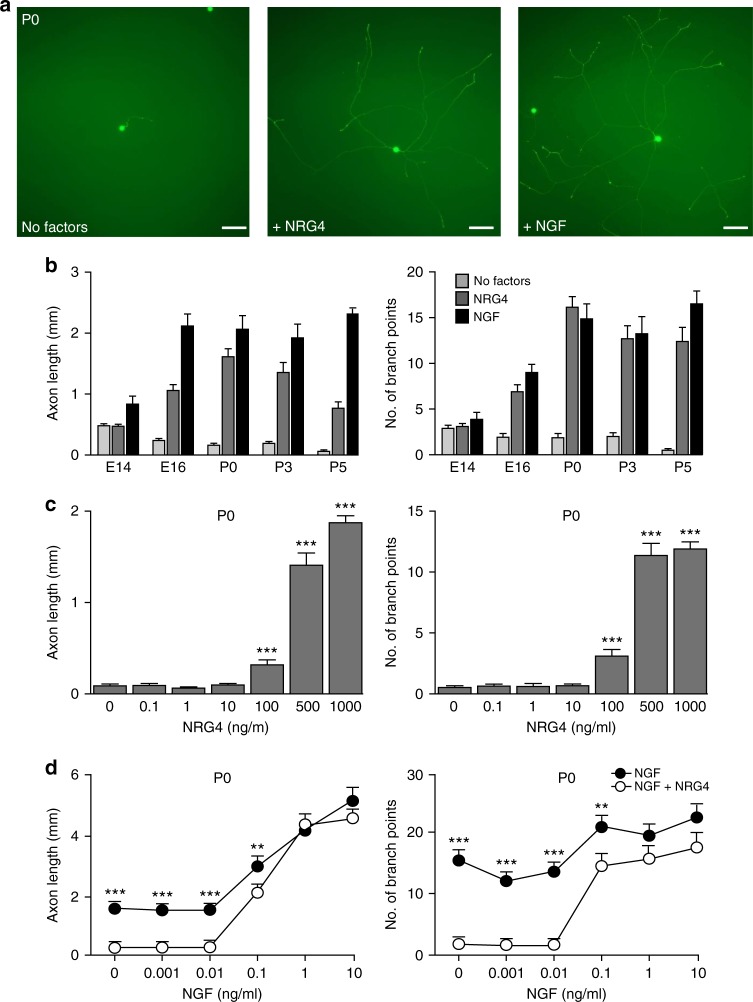
NRG4 promoted primary sympathetic neuron axonal growth and branching. **a** Representative images of P0 primary sympathetic neurons cultured for 24 h without factors (control) or with NRG4 or NGF, Scale bar, 50 µm. **b** Quantification of axon length and number of branch points in SCG neuron arbors at stage E14 to P5 cultured without factors or with either NRG4 or NGF. **c** Axon growth and branching dose responses of P0 SCG neurons to NRG4. **d** Axon growth and branching dose responses of P0 SCG neurons to NGF with and without 1 μg/ml NRG4. Mean ± s.e.m of data of >150 neurons per condition combined from 3 experiments. Mean ± s.e.m. are presented. *P < 0.05, **p < 0.01, ***p < 0.001 compared to 0 ng/ml NRG4 (**c**) and NGF conditions (**d**) using one-way (**c**) or two-way (**d**) ANOVA with Bonferroni–Holm post-hoc multiple comparison test

We then determined whether NRG4 influenced the growth and branching of sympathetic SCG neurons by treating them with either NRG4 or NGF. Like NGF, NRG4 promoted axon growth and branching (Fig. 4a, b). At the highest concentration used, NRG4 promoted branching as effectively as the maximally effective concentration of NGF and acted over a similar developmental time course as NGF. NRG4 was not as potent in promoting axon growth as NGF, and exerted its greatest effect at P0, whereas the axon growth-promoting effect of NGF plateaued at E16 (Fig. 4b). The concentration of NRG4 required to exert these effects was two orders of magnitude higher than the maximally effective concentration of NGF (Fig. 4c). There was no additive effect of NRG4 and NGF in combination (Fig. 4d), suggesting that these factors affect overlapping populations of SGC neurons. These results suggest that NRG4 promotes the growth and branching of sympathetic axons during the period when axons are growing and ramifying in their target tissues.

### AT BMP8b overexpression induced vascular remodeling

**Fig. 5:**
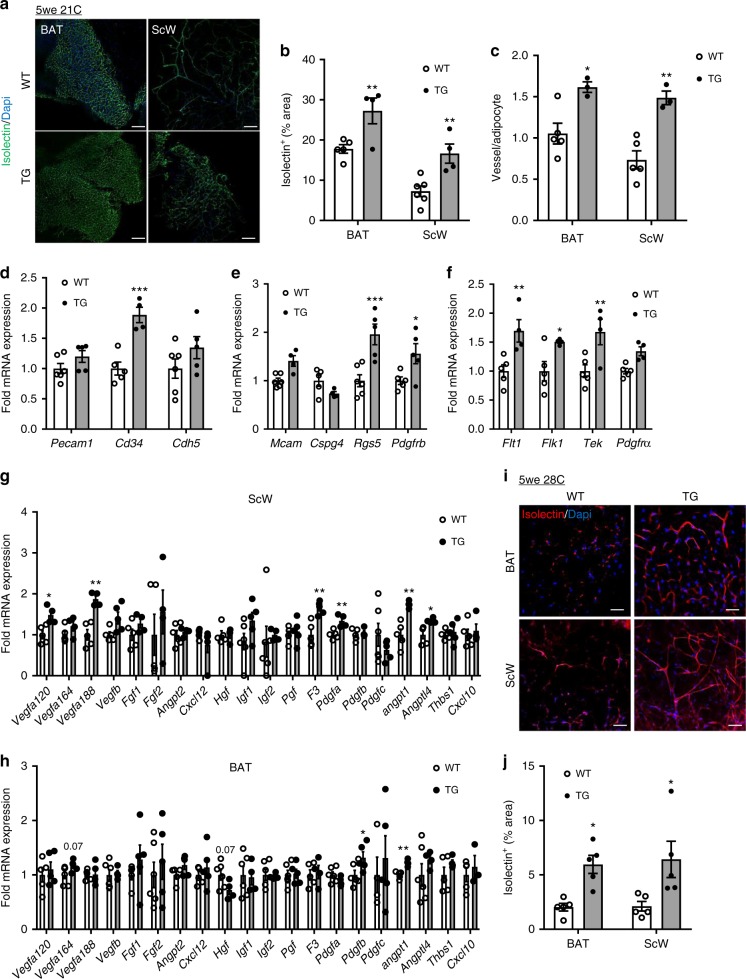
BMP8b overexpression induced remodeling of vasculature network. **a**, **b** Representative images of confocal analysis of Isolectin IB4 staining in BAT and ScW from 5-week-old WT and *Bmp8b* TG mice (**a**) and quantification of % of positive area (**b**). Scale bar, 100 µm. **c** Quantification of the number of vessels (isolectin^+^, caveolin-1^+^) per adipocytes (caveolin-1^+^, isolectin^-^) based on the representative images in Supplementary Figure 5a; *n* = 3–6. **d**–**f** Fold variation of mRNA levels of ECs (**d**), pericyte (**e**) markers and angiogenic factor receptors (**f**) in ScW from 4-week-old *Bmp8b* TG female mice, *n* = 4–6. Data are expressed in fold variation over control (4-week-old WT littermate). **g**, **h** Fold variation of mRNA levels of pro-angiogenic factors, factors promoting the maturity of newly made capillaries (*Angpt1, Angptl4*) and anti-angiogenic factors (*Thbs1,Cxcl10*) in ScW (**g**) and BAT (**h**) from 4-week-old *Bmp8b* TG female mice, *n* = 4–6. Data are expressed in fold variation over control (4-week-old WT littermate). **i**, **j** Representative images of confocal analysis isolectin staining in BAT and ScW from 5-week-old WT and *Bmp8b* TG female mice born and housed at 28 °C (**i**) and quantification (**j**) of % area. Scale bar, 100 µm. Mean ± s.e.m. are presented. *P < 0.05, **p < 0.01, compared to WT using two-way ANOVA with Sidak’s post-hoc multiple comparisons test (**b**, **c**, **j**); *p < 0.05, **p<0.01, ***p<0.001, compared to WT using multiple *t*-test with FDR determined using the two-stage linear step-up procedure of Benjamini, Krieger and Yekutieli (**d**–**h**)

Cold-induced recruitment of BAT requires a parallel expansion of the vascular network to perfuse the new brown adipocytes. BAT and ScW sections labeled revealed a significant increase in the vascular network (isolectin B4) associated with a higher number of vessels per adipocyte (ratio Isolectin B4/caveolin-1) in post-weaning *Bmp8b* TG mice, compared to WTs (Fig. 5a–c, Supplementary Figure 5a). Moreover, pericyte coverage (ratio NG2+/CD31+) was enhanced in *Bmp8b* TG mice indicating the enhanced functionality and stabilization of vascular networks in young *Bmp8b* TG mice^29^. No differences in vascularization network were observed in GnW (Supplementary Figure 5b-d). At the transcriptional level, angiogenesis-related factors were differentially up-regulated depending on the fat depot. Accordingly, endothelial (i.e., *cd34*) and pericyte (e.g., *pdgfrb*) markers and angiogenic factors/receptors, such as *vegf-a* and *angpt1* (Fig. 5d–h), were up-regulated in ScW of 4-week-old BMP8b TG mice and to a lesser extent in BAT (Supplementary Figure 5e-g). In support of a local regulatory effect, the ScW and BAT hyper-vascularization in *Bmp8b* TG mice was preserved when mice were born and housed at 28 °C (Fig. 5i, j).

Since pericytes share common markers, such as platelet-derived growth factor receptor-α (PDGFRα), with adipocyte progenitors^30,31^, we assessed by flow cytometry the number of adipose progenitors in the AT of Bmp8b TG mice. Our results show that the number of ScW adipose progenitors (gated as CD45^−^, CD31^−^, CD34^+^, PDGFRα^+^^32^) in *Bmp8b* TG mice were similar to WTs (Supplementary Figure 6). These results indicate that the beige adipocyte recruitment in the ScW from *Bmp8b* TG mice likely resulted from increased brown adipogenesis or adipocyte transdifferentiation rather than increased recruitment of progenitors. Chronic treatment with BMP8 during adipogenesis directly increased thermogenic and brown marker expression in brown (C57 brown cells) and to a lesser extent in white adipocytes (3T3-L1 cells). Some of these thermogenic genes were also induced following acute (24 h) BMP8 treatment on differentiated brown and white adipocytes (Supplementary Figure 7a-d).

### BMP8b pro-angiogenic effect requires adipocyte activation

**Fig. 6:**
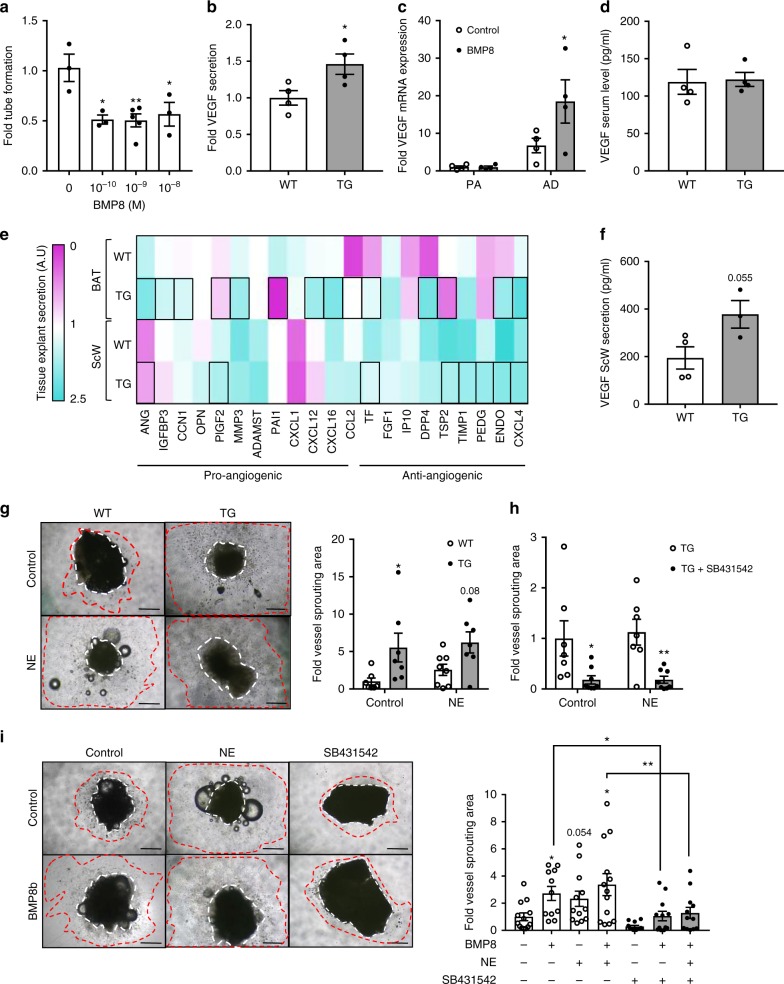
BMP8b induces a pro-angiogenic profile in adipocytes. **a** Effects of recombinant BMP8 on MCEC vascular tube formation (*n* = 3–5 experiments). The data shown are representative of one experiment performed in triplicate. **b** VEGF protein level secreted from mature adipocytes isolated from ScW of 10-week-old WT or *Bmp8b* TG mice (24 h secretion); *n* = 4. **c** mRNA levels of angiogenic factors in white adipocytes treated or not with recombinant BMP8 (100pM) before (PA, 24) and during (AD, 9 days) differentiation; *n* = 4. **d** VEGF protein in serum of 10-week-old WT and *Bmp8b* TG mice; *n* = 4. **e** Heat map presenting fold variation of protein levels of pro/anti-angiogenic factors secreted from ScW and BAT explants from 10-week-old WT and TG mice. Data from one experiment of angiogenic proteome array (Supplementary Figure 8d) on a pool of three independent experiments are presented. **f** VEGF protein level secreted from ScW explants of 10-week-old WT or *Bmp8b* TG mice (24 h secretion); *n* = 3–4. **g**–**i** Representative images of 10-week-old WT and *Bmp8b* TG (TG) mice ScW explants (**g**) or BMP8-treated explants (**i**) cultured on Matrigel without (Control) or with NE stimulation (10^–7 ^M) (**g**) and/or without (TG) or with (TG+SB431542) Alk7 antagonist (SB431542) for 10 days (**h**). Dotted orange lines encompass the explant; dotted red lines show the cell sprouting area. Quantification of cell sprouting area, *n* = 7–8. Scale bar, 500 µm. Mean ± s.e.m. are presented. *P < 0.05, **p<0.01, compared to 0 M BMP8 condition using one-way ANOVA with Sidak’s post-hoc multiple comparisons test (**a**, **c**, **i**), *p < 0.05, **p<0.01, compared to WT (**g**) or TG (**h**) using two-way ANOVA with Sidak’s post-hoc multiple comparisons test; *p < 0.05 compared to WT using a *t*-test (**b**)

To ascertain whether BMP8b promoted angiogenesis directly, we used the in vitro Matrigel angiogenesis assay^33^ and tested on a mouse cardiac endothelial cell whether recombinant BMP8b increased vascular tube formation. However, after 6 h of treatment with BMP8b, angiogenesis was suppressed (Fig. 6a), an effect unrelated to toxicity (Supplementary Figure 8a). This raised the possibility that, as for innervation, BMP8b might exert its pro-angiogenic effect indirectly by simulating the production of a pro-angiogenic factor(s). Consistently, we found that isolated mature ScW adipocytes of *Bmp8b* TG mice produced significantly higher VEGF level compared to those from WT mice (Fig. 6b). The pro-angiogenic role of mature white adipocytes was also validated in vitro by showing that BMP8 induced *Vegf* expression in differentiated adipocytes (Fig. 6c) and that conditioned media from adipocytes pre-treated with BMP8 enhanced tube formation in Matrigel (Supplementary Figure 8b). Importantly, circulating VEGF levels were not altered in *Bmp8b* TG mice (Fig. 6d), indicating a predominantly local effect of BMP8-induced VEGF production in the vascular remodeling of the ScW. Accordingly, VEGF release from ScW explants was increased by BMP8 treatment (Supplementary Figure 8c). Using an angiogenesis array approach to characterize the pro-angiogenic effect of BMP8b, we found that in addition to VEGF and PlGF2, BMP8 also increased (>1.3) the release of pro-angiogenic molecules such as CCN3, Proliferin, HGF and IGFBP3 from brown adipocytes (Supplementary Figure 8d, e).

Pro-angiogenic capacity of ScW explants from *Bmp8b* TG mice was validated using a similar angiogenesis array that showed higher levels of pro-angiogenic factors (e.g., VEGF, PlGF2) and decreased levels of anti-angiogenic factors (e.g., IP10, endostatin) compared to WTs (Fig. 6e, f, Supplementary Figure 8f). These results indicate that BMP8b might increase the balance between pro- and anti-angiogenic processes. We next evaluated the capacity of ScW explants for vascular sprouting on Matrigel, assessing angiogenesis in an environment composed of multiple cell types, maintaining the endothelial cell (EC)/adipocyte cross-talk^34^. Vascular sprouting was increased in ScW explants from *Bmp8b* TG mice compared to WT independently of the presence of NE (Fig. 6g). The dependency on BMP signaling was confirmed as treatment of *Bmp8b* TG ScW explants with the BMP8b receptor Alk7 antagonist SB431542^17^ completely blocked vascular sprouting (Fig. 6h). Furthermore, chronic treatment of ScW explants in Matrigel with recombinant BMP8 increased sprouting capacity in a similar way than in response to NE, whereas sprouting was reversed in the presence of SB431542 (Fig. 6i). Taken together, these results support an autocrine role for BMP8b regulating vascular remodeling in ScW by increasing the pro-angiogenic activity of the adipocytes.

### Integration of angiogenic and innervation in BMP8b TG mice

**Fig. 7:**
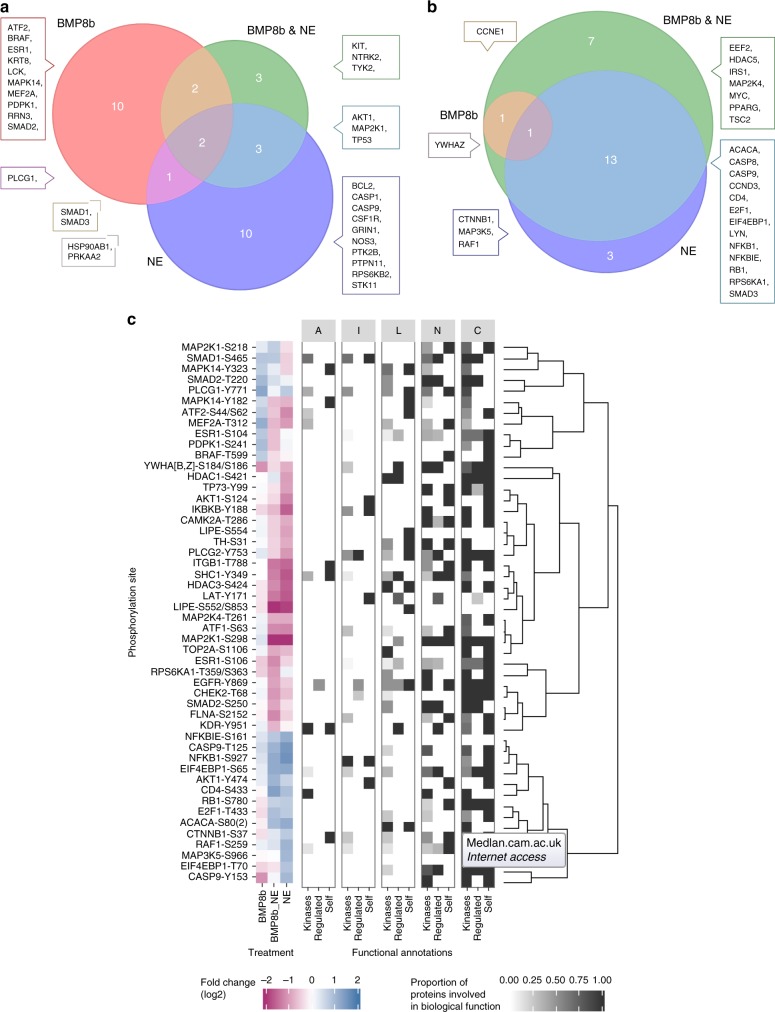
Phophoproteomic array analysis of BMP8b signaling in brown adipocytes. Brown differentiated adipocytes were treated for 30 min with human recombinant BMP8 (100 pM) or NE (75 nM) or combination of both, prior to phosphoproteome analysis. **a**, **b** Venn diagrams showing proteins with fold change values above 2 (twice more (**a**) or half less (**b**) phosphorylated) on any of their phosphosites between treatments. Proteins are represented by standard human gene names. The number of proteins in each field is proportional with the area of the field and shown by numbers. **a** Positively regulated proteins according to fold change signs adjusted by the known effect signs. Phosphosites without known effect signs were not considered here and at (**b**). **b** Inhibited proteins with similar sign adjustment. **c** Heat map representing sign adjusted fold changes and functional annotations of the top 50 phosphosites in BMP8, NE and BMP8 and NE treated brown adipocytes. Cells in the leftmost frame show log2 fold change values with signs adjusted by their known effects. Green, white and red corresponds to inhibition, no change and stimulation, respectively. Clustering and dendrogram constructed with Euclidean distance and complete linkage. Sites for which no effect sign is known from PhosphpSitePlus are not shown here. On the middle frames are shown the functional annotations mapped from the Gene Ontology for the substrates (named as “self”), their kinases (name as “kinases”) and their downstream regulated interaction partners (named as “regulated”). Shades of gray of each cell correspond to the proportion of all interacting proteins annotated with at least one term in the group. A angiogenesis, I inflammation, L lipid metabolism and thermogenic activity, N neurogenesis, C cell cycle and survival

To identify BMP8b downstream effectors and points of cross-talk between signaling pathways regulating neuro-vascular remodeling, we performed a phosphoproteomics-based study using a phospho-antibody microarray on brown differentiated adipocytes stimulated with BMP8 and/or NE. We used a (i) candidate-based approach (i.e., biological process terms determined in Gene Ontology (GO)^35^ relevant for tissue remodeling: angiogenesis; cell cycle and survival, inflammation, lipid metabolism and thermogenic activity and neurogenesis), (ii) pathway enrichment analysis using the ”piano” R package^36^ (Supplementary Figure 9a, b, Supplementary Data 1-4, Supplementary Software). We found 31 and 25 proteins that were respectively significantly up- or down-regulated by the different treatments (Fig. 7a, b). Venn diagrams confirmed the expected role of SMADs in BMP8b signaling; SMAD 1 and 3 being activated only by BMP8 stimulation. Notably, activation of AKT1-, MAP2K1- and TP53-dependent pathways, involved in regulation of neurogenesis and cell cycle/survival, appeared to be NE dependent. We also observed that phosphosites up-regulated by BMP8 were mainly stimulatory. Surprisingly, combined treatment of NE+BMP8 led to down-regulation of 7 proteins, among them HDAC5 and EEF2 (neurogenesis) and IRS1, PPARG (lipid metabolism and thermogenesis), suggesting a potential feedback regulation preventing excessive stimulation. BMP8 and NE conditions independently phosphorylated and activated 10 protein candidates and only shared one, showing that the role of BMP8b amplifying NE response might not be cumulative to NE but independent, activating additional pathways. Interestingly, BMP8 specifically up-regulated proteins directly associated (named as “self”) with thermogenesis (ATF2, BRAAF, MAPK14), neurogenesis (BRAAF, MEF2A), angiogenesis (MAPK14) and cell survival (ATF2, ESR1, PDPK1) (Fig. 7c). Among the 10 proteins up-regulated by BMP8, some were related to other pathways. For instance, ATF2 and MEF2A were also associated with angiogenesis and thermogenesis because their upstream kinases were described to regulate or be regulated by these pathways. Similarly, ESR1 was also related to thermogenesis and neurogenesis because the functions assigned to its downstream proteins were regulated by the phosphorylation events.

**Fig. 8:**
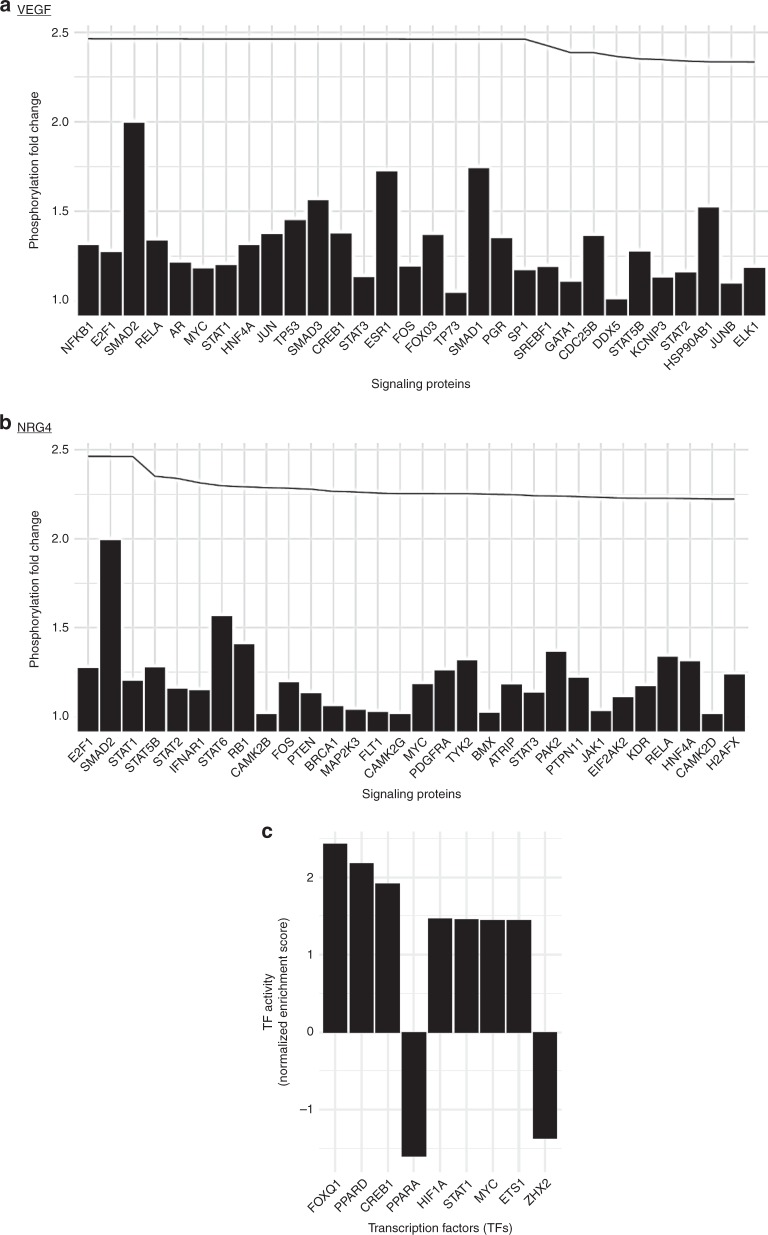
BMP8b-dependent signaling proteins involved in *vegfa* and *nrg4* regulation. **a**, **b** BMP8-induced phosphorylation state fold change of signaling proteins ordered by network distance to the transcription factors regulating *vegfa* (**a**) and *nrg4* (**b**). Bars show the fold changes of the most affected phosphorylation site on each protein, 1 corresponding to no change while, for example, 2 meaning the proportion of phosphorylate proteins doubled upon BMP8 treatment. The top line shows the log scale proximity of the signaling proteins to the transcription factors, from the closest on the left, distance increases to the right. **c** Estimated activities of transcription factors regulating *vegfa* in BAT from 5-week-old *bmp8b* TG mice estimated from expression data. Positive and negative values correspond to up-regulated and down-regulated TFs, respectively. Ordered by *p* value from the lowest on the left, showing only *p* value < 0.2

We then integrated these phosphoproteomics data with the gene expression profile of the BAT from *Bmp8b* TG mice, to investigate by which signaling pathways BMP8b could regulate neuro-angiogenic factors, notably *vegf* and *nrg4* expression. We performed a network analysis in which we collected the transcription factors (TFs) regulating the expression of all the genes we analyzed in the BAT from 5 weeks-old *Bmp8b* TG mice and calculated the distances in this network from each of the proteins measured on the phosphoassay to all TFs. We identified a top list of signaling proteins with phosphorylation state affected by BMP8 showing close network distance to the TFs regulating *vegf* and *nrg4* (Fig. 8a, b). At *vegfa*, we also identified 4 TFs, SMAD2, SMAD1, ESR1 and SMAD3, showing larger than 1.5-fold increase in their phosphorylation upon BMP8 treatment and directly regulating *vegfa* transcription (Fig. 8c). Regarding *nrg4* signaling, SMAD2 and STAT1 showed increased phosphorylation upon BMP8 treatment constituting as a potential transcriptional regulator of *nrg4* expression. Taken together, these results validated the role for BMP8b, independent of NE, in brown adipocytes mediating the remodeling of the neuro-vascular networks in BAT and beige tissues. Moreover, these data also highlighted new molecular candidates and signaling pathways driving brown thermogenesis and tissue remodeling.

We next investigated whether neurogenic, thermogenic and angiogenic factors were sequentially regulated and which processes preceded or followed the others. First, we mapped and compared the gene expression profile in BAT and ScW between two close time point, 4-week-old and 5-week-old mice (Supplementary Figure 10). Our results did not show clear temporal differences in the differential regulation of thermogenic, angiogenic and neurogenic factors, which were all mostly up-regulated at 4-week-old in BAT and ScW. These results support the tight co-expression and co-regulation of thermogenic, angiogenic and neurogenic genes.

Regarding the influence of the thermogenic state, we observed that *ucp-1* was massively up-regulated in ScW from 4- and 5-week-old *bmp8b* TG mice compared to WT. This raised the possibility that neuro-vascularization observed in TG required UCP1-dependent thermogenic metabolism in adipose tissues, which subsequently could promote innervation and angiogenesis. Therefore, we exploited a dataset performed in UCP1-deficient (*ucp1*^*–/–*^*)* mice acclimated to 4 °C, from Keipert et al.^37^ (GSE99412) (Supplementary Figure 11 a-c) and investigated the UCP1 dependence of the regulation of *bmp8b* and neurogenic/angiogenic factors. In this study, Keipert et al.^37^ demonstrated that cold-induced browning of the ScW was still functional in *ucp-1*^–/–^ mice^37^. We observed that in addition to thermogenic factors, both *bmp*8b and *nrg*4 were up-regulated (Supplementary Figure 11d). Moreover, despite *vegf* not being significantly affected, we observed a large enrichment of pro-angiogenic molecules in the top 30 significantly up- and down-regulated genes (false discovery rate (FDR)-adjusted *p* < 0.05) (Supplementary Figure 11e). These results are compatible with cold-induced BMP8b triggering adipose neurogenesis and angiogenesis independently from UCP-dependent mechanisms.

Unlike WAT innervation, vascularization in *Bmp8b*^–/–^ and *Bmp8b* TG mice did not mirror each other. While ScW explants from adult *Bmp8b*^*–/–*^ mice displayed a tendency to decreased vascular sprouting (Supplementary Figure 12a), 5-week-old *Bmp8b*^*–/–*^ mice did not show a significant decrease in vascular density (Supplementary Figure 12b). This led us to hypothesize that the WAT vascularization phenotype in Bmp8b TG mice may be part of a homeostatic response triggered by augmented functional requirements and might require a primary increase in innervation in response to cold. Thus, if true, the need for increased vascular remodeling at room temperature might not be required if the innervation was decreased. To test this hypothesis, *Bmp8b*^–/–^ mice were acclimated to 4 °C for 1 week and the neuro-vascular gene expression profile was screened in BAT and ScW (Supplementary Figure 12c, d). As anticipated, *ucp-1* and neurogenesis-related genes were down-regulated in *Bmp8b*^–/–^ mice compared to WT mice. This was followed by the down-regulation of *vegf*, the vascularization marker *cd34* and pericyte markers (*rgs5* and *pdgfβ*). These results confirm the relevance of BMP8b mediating adipose tissue angiogenesis remodeling under condition of increased thermogenic demand, since in the absence of BMP8b the cold exposure-induced pro-angiogenic response was prevented.

## Discussion

**Fig. 9:**
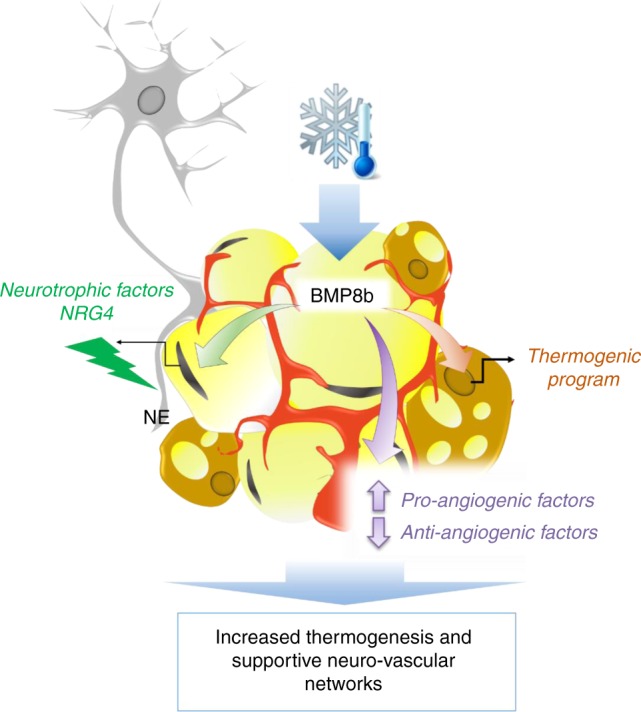
Summary of proposed roles of Bmp8b in AT. BMP8b overexpression in adipocytes and subsequent hyper-activation of the sympathetic tone induce the recruitment of thermogenic beige adipocytes in ScW which are accompanied by the expansion of the sympathetic nervous and vascular networks. BMP8b notably promotes outgrowth and branching of sympathetic nerve axons, through NRG4 production, and the production of new capillaries by regulating the function of vascular cells and production of angiogenic factors

We provide evidence that BMP8b enhances the thermogenic capacity of BAT and beige tissues by increasing adipocyte production of NRG4 and angiogenic factors that coordinately remodel thermogenic ancillary networks (vascular and nervous networks) (Fig. 9). This is the first study identifying adipocyte-secreted BMP8b as a potential regulator of neuro-vascular processes, essential to couple adrenergic responsiveness, adipose tissue remodeling and efficient thermogenesis.

From the initial insight that the thermogenic markers of canonical BAT were not different between the two genotypes, we rationalized that the increased thermogenic capacity observed in the *Bmp8b* TG mice may depend, at least in part, on the BAT-like phenotype observed in their ScW. Also the fact that the increased expression of thermogenic and browning genes in *Bmp8b* TG mice occurred early in the post-weaning phase, when mice are more vulnerable to low temperatures, suggested the physiological relevance of BMP8b mediating the adaptation to cold demands. Another important insight about the BMP8b-mediated increase in NE response came from the histology of the BAT and ScW of the *Bmp8b* TG mice. We observed increased Scw/BAT sympathetic innervation density. Further analysis may require sophisticated techniques (e.g., two-photon microscopy) allowing powerful imaging of sympathetic projection density^38,39^ avoiding the occasional spot-like appearance of TH fibers resulting from lateral sections in immunohistochemistry analysis.

BMP8b itself did not promote axonal growth directly but required the positive regulation of *Nrg4* expression and its secretion by adipocytes. These data indicated that adipocyte-derived NRG4 may have an essential role in regulating BAT innervation in addition to its “batokine” peripheral metabolic effect, previously reported to act on liver^16^. The importance of NRG4 is also highlighted by the fact that NRG4 is the only neuregulin family member reportedly expressed in WAT and BAT, and by white and brown mature adipocytes^16^. The fact that *Nrg4* gene expression is physiologically up-regulated by cold exposure in BAT and ScW in parallel with BMP8b further emphasizes its physiological relevance in cold-induced nervous network remodeling^15^.

While investigating how BMP8b overxpression lead to ScW browning, we found that BMP8b increased ScW vascularization in vivo and ex vivo. As for innervation, this effect was due to BMP8b promoting a pro-angiogenic transcriptional and secretory profile in adipocytes rather than through a direct effect on endothelial cells. Despite VEGF being an important factor, it was not the only angiogenic molecules regulated by BMP8b. We showed a switch in the balance of angiogenic/anti-angiogenic molecules toward a pro-angiogenic activity in the adipocytes overexpressing/treated with BMP8b. Given that inhibition of VEGF pathway has been described to prevent BAT/browning activation^12–14^, selective ablation (pharmacologically or genetically) of VEGF in *Bmp8b* TG mice may provide further support to this conclusions.

Taken together, these data indicate that whereas BMP8b is not essential for the development and maintenance of the basal vascular network in AT, the induction of BMP8b, typically observed during cold-induced adrenergic stimulation, can be considered as a regulator of the vascular remodeling in BAT and ScW required to match increased thermogenic demands.

Our data strongly support the local remodeling effect of BMP8b in WAT given that active remodeling is maintained in mice born and housed at 28 °C. This is an important experimental detail, given that 28 °C corresponds to the environmental temperature at which the SNS input is at minimal level. At 28 °C, the adipose tissue of the BMP8b transgenic mouse still displays a robust increase in neuro-vascularization, scW browning and thermogenic capacity, which is unlikely to result from the enhancement of the remaining small adrenergic signal provided by the SNS. Our data demonstrate this is a cell- and tissue-autonomous effect since ScW explants from *Bmp8b* TG or from WT mice treated with BMP8b displayed increased vascular sprouting and differential secretion of angiogenesis-related factors compared to WT mice. Once again, these effects cannot be ascribed to central effect (since these cells were cultured ex vivo) and are not influenced by exposure to adrenergic agonist (NE). Its independence from central nervous system input is further supported by the lack of changes in efferent SNS activity in *Bmp8b* TG mice.

Our proteomic results indicate that rather than simply reinforcing the NE-dependent signaling pathways, BMP8b is a thermogenic regulator whose function does not exclusively depend on the activation of the SNS but on its direct trophic action to supporting structural networks. This model of regulation has the advantage of being regulated at local level by the target organ response and not exclusively and/or primarily by the sympathetic inflow. This biological strategy should efficiently titrate the local adaptation required to fulfill the functional thermogenic demands more accurately.

We used an in vivo transgenic approach to model the effects of the induction of BMP8b in WAT/BAT typically observed during cold exposure and identify potential triggers controlling adipose tissue remodeling in response to cold. The relevance and autonomy of these mechanisms has been subsequently validated using purposely designed experiments. We rationalized that instead of using adiponectin or UCP-1 to drive transgene expression, the aP2 promoter offered the advantage of driving expression not only in mature adipocytes but also in progenitors, a feature that is important considering its relevance in the process of beige cell recruitment^40^. However, we were aware that this transgenic approach has intrinsic limitations related to persistent overexpression from embryogenesis, the achieved level of transgene expression and/or the possibility of ectopic confounding non-adipose effects. Our observations could be refined with inducible models to control the expression of Bmp8b either at 5 weeks or 12 weeks of age which would further confirm the role of BMP8b as a potential regulator of AT neuro-vascular remodeling.

Our results have shown that beige cells, despite their early development and apparent disappearance during adulthood, retain their hyper-responsiveness to adrenergic stimulation in adult mice, behaving as “*dormant*” beige cells. This time profile is potentially of interest as it may define a critical time interval, early in life, where the contribution of BMP8b to the development and recruitment of the beige cell pool is important. This time window was also suggested by Contreras et al.^41^ who showed that beige cell recruitment is particularly active in ScW of mice between 10 and 30 days postnatal. After this early time window, developed beige cells remain functionally “dormant”, but ready to be awoken later on in response to cold stress or to β3-adrenergic agonists. Our results indicate that maintenance of these latent beige adipocytes in white depots is dependent on BMP8b and that these latent cells are functionally important by enabling a quick and efficient thermogenic response under challenging environmental or nutritional conditions^41^.

In summary, we have shown that the increase in BMP8b secretion by adipocytes under conditions of increased thermogenic demand, such as cold exposure or HFD, might contribute for the efficient remodeling required for BAT/beige recruitment and function. Most browning models described in the literature are based on targeting late effectors of thermogenic, angiogenic factors or the SNS^14,42,43^. BMP8b is an upstream adipocyte-derived regulator that optimizes neuro-vascular remodeling coupled to tissue thermogenic demands. Thus, by targeting Bmp8b, and acting upstream of these three important pathways, it should be feasible to control the efficient coordination of adipocyte thermogenesis, angiogenesis and innervation. The evidence that BMP8b-mediated signaling may couple browning and optimization of the supporting neuro-vascular network provides the rationale for a new and safe approach to the treatment of the obese patient, addressing the problem of previous anti-obesity therapy-based adrenergic stimulation, discontinued because of associated hypertension and/or tachycardia. This is not only based on optimizing the efficacy of BAT-specific adrenergic agonists in terms of activating and recruiting BAT or beige fat, but also in optimizing the ancillary systems of angiogenesis and neurogenesis, which may decrease the side effects inherent to thermogenic strategies exclusively based on promoting adrenergic stimulation.

## Methods

The key resources including genetically modified mice models, cell lines, reagents, antibodies and software used in the present study are indicated in the Supplementary Table 1.

### Animals

Unless otherwise stated, all data are from work on females. C57Bl6/J mice were purchased from Charles River. *Bmp8b* ^–/–^ mice were generated as previously described^44^ on a C57Bl6/J background and were compared to wild-type littermates. Unless stated, mice were housed in a temperature-controlled room (21 °C) with a 12 h light/dark cycle with free access to diet and water. Regarding *Bmp8b* TG mice, WT SV129*C57Bl/6J mixed strain mice were engineered to express the murine *Bmp8b* gene from the apolipoprotein 2 (*Fabp4*) promoter. Mice were backcrossed until whole genome analysis confirmed that they were >98% C57Bl/6J genotype. For each experiment, the age of the mice is stated in the figure legends. This research has been regulated under the Animals (Scientific Procedures) Act 1986 Amendment Regulations 2012 following ethical review by the University of Cambridge Animal Welfare and Ethical Review Body (AWERB) and all ethical regulations were complied with. The UK Home Office, USC Bioethics Committee and the University of Iowa Animal Research Committee approved all animal procedures.

### Diet and temperature studies

Diets for animal studies included standard chow (sodium dodecyl sulfate (SDS), 10% calories from lipid) and an HFD (D12451, Research Diets, 45% calories from lipid). Standard chow or HFD was administered ad libitum to animals from weaning until indicated. Studies in conditions approaching thermoneutrality involved (1) mice born and housed at 28 °C or (2) mice housed at 28 °C for 3 weeks. Fasting consisted of removing food for 24 h, and refeeding involved fasting followed by replacement of food for 24 h. Fat and lean masses were calculated by time-domain nuclear magnetic resonance using a minispec Live Mice Analyzer LF50 (Bruker).

### Energy expenditure and maximum thermogenic capacity

Energy expenditure (EE) and respiratory quotient (RQ) were calculated from data gathered from single-housed mice fed ad libitum by a monitoring system based on home cages (Ideas Studio) designed by Dr. Peter Murgatroyd. The monitoring system calculates oxygen and carbon dioxide concentrations at 18-min intervals. EE was calculated from oxygen consumption and carbon dioxide production measured over a 48 h period using indirect calorimetry with the Elia and Livesey constants for TQ. Maximum thermogenic capacity of mice was assessed by indirect calorimetry, performed in an Oxymax calorimetry chamber which had a 2.7-liter capacity. Temperature within calorimetric chambers was continuously monitored and fixed at 30 °C. Oxygen consumption and carbon dioxide production were measured using a custom built oxygen and carbon dioxide monitoring system built by P. Murgatroyd. Airflow rates were set at 400 ml/min. Measurements of oxygen concentration and carbon dioxide concentration in room air and air leaving each cage were measured every 4 min. Mice were placed in the calorimetric chamber following anesthesia by intra-peritoneal injection of sodium pentobarbital (60 mg/kg). Baseline gas exchange was recorded once steady state was achieved (at least 3 consecutive stable measurements). Mice were then injected with 1 mg/kg noradrenaline bitartrate (Sigma, UK) subcutaneously and returned to the calorimetry chamber. Maximal oxygen consumption rates were typically achieved within 12–16 min post injection and were defined either as 3 stable consecutive readings or when oxygen consumption rates began to fall. Energy expenditure was calculated from VO2 and VCO2 using the modified Weir equation.

### Cell culture and differentiation

The immortalized brown adipocyte (C57BAT) cell line was a gift from the laboratory of Johannes Klein and was generated as previously described^17^. By day 8 post induction, cells were defined as differentiated if they appeared healthy and lipid replete. T3 and insulin were removed from the media 24 h prior to any treatment with these molecules. 3T3-L1 Cells were differentiated into adipocytes (day 9) accordingly to the protocol described by Roberts et al.^45^.

### Catecholamine measurement

Adipose tissues (BAT, ScW, GnW), skeletal muscle and serum were collected from 12-week-old *bmp8b* WT and TG mice kept at 21 °C or from 5-week-old *bmp8b* WT and TG mice born and housed at 28 °C. Tissue extract and serum were prepared in 2% perchloric acid (PCA) (20 mg of tissue/20 μl serum per 200 μl 2% PCA) and analyzed for NE content using reversed-phase high performance liquid chromatography (HPLC). The mobile phase was prepared using HPLC grade reagents and consisted of citric acid (31.9 g/l), sodium acetate (3.3 g/l), sodium octane sulphonic acid (460 mg/l), methanol (10%), adjusted to pH 3.7. Separation was achieved using a Hyperclone® 5μ BOS C18 analytical column (Phenomenex, UK) and a flow rate of 0. 75 ml/min. Analytes were detected electrochemically (Coulochem II, ESA, Inc., Chelmsford, MA, USA) with E1 –200 mV and E2 +250 mV. Chromatograms were acquired and integrated using the Chromelean Chromatography Data System (V6.2, Dionex, UK).

### Lipolysis assays

Brown and white mature adipocytes were isolated from ScW of 10–12-week-old *Bmp8b* WT and TG mice and embedded in a hydrogel (PuraMatrix®, Corning) for 24 h prior to adrenergic antagonist stimulation as described in ref. ^46^. Adipocytes/hydrogel were then incubated in Krebs–Ringer bicarbonate buffer with or without NE (75 nM) for 4 h at 37 °C. Glycerol was measured as an index of lipolysis by using free glycerol reagent (Sigma) against a glycerol standard curve.

### Immortalized mouse cardiac endothelial cells

Mouse cardiac endothelial cells (MCECs) were maintained in 5% Dulbecco’s modified Eagle's medium without sodium pyruvate with 5% heat inactivated fetal bovine serum (FBS), 20 mM HEPES, 100 units/ml penicillin, 100 μg/ml streptomycin and 20 mM l-glutamine (“MCEC growth media”) at 37 °C in 5% CO_2_ in flasks pre-coated with 0.02% gelatin.

### Matrigel assays

Pro-angiogenic capacity of BMP8 recombinant protein was assessed using the in vitro Matrigel angiogenesis assay^33^. MCECs were seeded at a density of 15,000 cells per well (96-well plate) pre-coated with 75 μl of Matrigel™-GF reduced (BD Biosciences) and cultured in MCEC growth media without FBS in the presence or not of BMP8 recombinant protein directly or conditioned medium from BMP8-treated adipocytes. After 8 h of incubation, phase-contrast images of the totality of the wells were taken with an Olympus CKX41 inverted microscope coupled to a DP20 digital camera. The total branching points of the network structures were quantified blind by two independent experimenters.

### Primary sympathetic neurons

NGF-dependent sympathetic neurons of the SCG of neonatal mice, at stages from E14 to P5, were removed and digested in 0.05% trypsin (Worthington) in Hanks' balanced salt solution at 37 °C for 30 min. SCGs were washed twice with 10% heat-inactivated horse serum (Gibco) in F-12 medium (Gibco), and then dissociated mechanically. Cells were plated on 35 mm dishes with 4 mini-wells at a density of approximately 100 cells per well; wells were pre-coated with poly-ornithine (Sigma) overnight and laminin (20 μg/ml; Sigma). Cells were maintained overnight at 37 °C in 5% CO_2_ for 18 h in growth medium supplemented as stated in the figure legends. Growth medium was F-14 media (JRH Biosciences) with 1.176 mg/ml sodium hydrogen bicarbonate, 200 units/ml penicillin, 200 μg/ml streptomycin, 2 mM l-glutamine, and 2% Albumax—a bovine serum albumin (BSA) solution supplemented with progesterone (60 μg/ml), putrecine (16 μg/ml), l-thyroxine (400 ng/ml), sodium selenite (38 ng/ml) and triiodothyronine (340 ng/ml) (all from Sigma). Cultures were treated with either BMP8 or NGF.Controls received neither factor. Apoptosis was prevented in cultures without NGF by the pancaspase inhibitor Boc^_^D^_^FMK. Individual SCG neurons were visualized by Calcein-AM (Invitrogen) staining and imaged blind by fluorescent microscopy (Zeiss Axiovert 200) after 24 h, and total neurite length and number of branches in the neurite arbors were quantified. All neurites in these short-term SCG cultures have the characteristics of axons^47^. Growth was assessed by Sholl analysis as described previously^48^.

### Explants for conditioned medium

Approximately 100 mg of freshly dissected ScW from 10-week-old WT or *Bmp8b* TG mice was incubated for 1 h at 37 °C in 5% CO_2_ in MCEC growth media without FBS supplemented (1 ml media per 100 mg of ScW). Subsequently, ScW was cut into fine pieces and incubated for 24 h at 37 °C in 5% CO_2_ in MCEC growth media (1 ml media per 100 mg of AT).

### Explants for assaying angiogenic growth

Wells of a 96-well plate were pre-coated with 40 μl of Matrigel, into which freshly dissected ScW fragments from 10–12-week-old WT or *Bmp8b* TG mice were embedded, and another 40 μl of Matrigel was added over the ScW fragment. Then, 100 μl of MCEC growth media was added, supplemented as indicated in the figure legend. Explants were maintained at 37 °C in 5% CO_2_ for 10 days, after which phase-contrast images were taken with an Olympus CKX41 inverted microscope coupled to a DP20 digital camera. Angiogenic outgrowth was quantified.

### Array-based detection of angiogenic factors

The Proteome ProfilerTM Mouse Angiogenesis Array Kit (R&D Systems, USA, Catalog Number: ARRY 015) was employed to screen for the presence of 53 soluble angiogenesis-related factors in the conditioned media from C57 brown adipocytes and BAT/ScW explants from *bmp8b* mice, according to the manufacturer’s instructions. Briefly, the angiogenesis antibody membrane was incubated for 1 h with an array blocking buffer prior incubation overnight at 4 °C on an orbital shaker with 1.5 ml of conditioned media. The membrane was then washed and incubated fpr 30 min with streptavidin-horseradish peroxidase solution. Membranes were exposed digitally using the ChemiDoc MP System (Bio-Rad), and spots were quantified using the ImageJ software. One condition corresponds to a pool of conditioned medium from four independent experiments. All the arrays were measured three times; each spots were normalized to the positives controls. The results are presented in a heat map and considered relevant when the fold variation was >1.3 or <0.6.

### ELISA assays

NRG4 protein concentration was measured using an ELISA kit for murine NRG4 (Catlag Medsystems, SEC174Mu) in conditioned media (from which debris was removed by centrifugation), serum and tissue (homogenized in phosphate-buffered saline (PBS)) according to the manufacturer’s instructions. A standard curve was prepared according to the manufacturer’s instructions, and the value associated with an unconditioned media blank was subtracted from that of conditioned media. VEGF-A protein concentration was measured using an ELISA kit for murine VEGF-A (R&D Systems) in cell conditioned media (from which debris was removed by centrifugation) according to the manufacturer’s instructions. A standard curve was prepared in unconditioned media.

### Whole-tissue immunofluorescence

AT samples were fixed in 4% paraformaldehyde for 1 h at room temperature and then transferred to PBS, in which they were stored at 4 °C until immunofluorescence analysis. Tissues were blocked in 1 M glycine and then PBS with 3% BSA and 0.1% Triton X-100. Samples were then incubated with the appropriate primary antibody or with fluorescein isothiocyanate-conjugated Isolectin B4 (Sigma) overnight at 4 °C. Bound primary antibody was detected if necessary after a further blocking step in PBS with 3% BSA using secondary antibodies conjugated to Alexa Fluor® dyes (488 and 568). Samples were mounted using VECTASHIELD® mounting medium with DAPI (4′,6-diamidino-2-phenylindole) and imaged blind using Zeiss LSM 510 Meta Confocal microscope with LSM 3D software (Carl Zeiss). At least three images per AT sample per stain were used for quantification, which was also performed blind. Cd31^+^, Ng2^+^ and isolectin^+^ areas were calculated using ImageJ software. The number of vessels per adipocyte was calculated from tissue stained for Isolectin (vessels) and caveolin (vessels and adipocytes). The number of pericytes per EC (pericytes coverage) was calculated by manually counting the number of Cd31^+^ and Ng2^+^ cells in a field of view, and then counting how many were in contact with Cd31^+^ cells.

### Tyrosine hydroxylase immunostaining

Fixed AT samples were dehydrated, cleared and paraffin embedded. TH immunoreactivity was determined by DAB immunohistochemistry using 3 μm sections from 3 different levels (100 μm apart) per sample. Briefly, sections were dewaxed and endogenous peroxidase blocked by incubating in 3% HO_2_ in methanol. Sections were then blocked in 3% rabbit serum and incubated overnight at 4 °C with anti-TH antibody (1/200) diluted in PBS. Bound primary antibody was detected using a biotinylated anti-sheep IgG secondary antibody, ABC reagent (Vector) and DAB Substrate kit (Sigma-Aldrich) according to the manufacturers’ instructions. Finally, sections were counterstained with hematoxylin. Parenchymal TH-positive fibers were analyzed by live count command in Lucia Imaging for image analysis (version 4.82, Nikon Instruments, Florence, Italy). The area of TH-positive nerve fibers was measured using images randomly captured with a Nikon E600 Eclipse microscope with camera. Ten areas from interscapular BAT were analyzed in each section. In inguinal ScW, 10 random areas were analyzed in each section. Areas of pure white adipocytes (larger, unilocular) and of pure beige adipocytes (smaller, multilocular) were analyzed separately.

### RNA extraction and real-time PCR

RNA from cells and from SCGs was harvested and purified using QiaShredder and RNeasy Mini columns (Qiagen) according to the manufacturer’s instructions. RNA was harvested from frozen tissue using RNA-STAT-60TM (AMS Bio), and purified by chloroform extraction and isopropanol precipitation.

Complementary DNA (cDNA) was generated in a 20 μl reaction as follows: 500 ng RNA was added to 4 μl 5x M-MLV reverse transcriptase master mix (Promega) with 2.5 mM MgCl_2_, 1.25 mM dNTPs, and 5 μg/ml random hexamers, and incubated for 5 min at 65 ^o^C before being transferred directly to ice. After the addition of 1 μl M-MLV reverse transcriptase, the reaction was incubated at 37 °C for 1 h and 95 °C for 5 min. cDNA was diluted 75-fold.

Quantitative reverse transcription-polymerase chain reaction (qRT-PCR) was performed in a 12.2 μl reaction with 5 μl of diluted cDNA using TaqMan (Applied Biosystems) primers and probes or SYBR green reagent (Applied Biosystems) according to the manufacturer’s instructions. Primer sequences are described in Supplementary Table 2. Reactions were run in duplicate for each sample and quantified using the ABI Prism 7900 sequence detection system (Applied Biosystems). Duplicates were checked for reproducibility, and then averaged. Product specificity was determined using a dissociation curve for SYBR green reactions. A standard curve generated from a pool of all cDNA samples was used for quantification. The expression of genes of interest was normalized using BestKeeper©7 to the geometric average four housekeeping genes (18s, 36b4, β-actin and B2m).

### Western blotting

Proteins were extracted from tissue in lysis buffer (20 mM Tris-HCl, 150 mM NaCl, 1 mM EDTA, 1 mM EDTA, 1% Triton X-100, pH 7.5) with added protease inhibitor (Roche) and phosphatase inhibitor (Roche) cocktails. Debris and fat were cleared from lysates by centrifugation. Protein concentration was determined by Dc Protein assay (Bio- Rad). After dilution in Laemmli buffer with 0.5% 2-mercaptoethanol, 30 μg protein was separated by electrophoresis in a NuPAGETM SDS-polyacrylamide gels (Invitrogen) and transferred to a nitrocellulose membrane using the iBlot® Dry Blotting System (Invitrogen). Membranes were blocked for 1 h in 5% milk protein or 5% BSA in tris-buffered saline with 0.05% Tween at room temperature, and incubated overnight at 4 °C with the appropriate primary antibody (Supplementary Table 1). Bound primary antibodies were detected using peroxidase-coupled secondary antibodies and enhanced chemiluminescence (Millipore). Blots were exposed digitally using the ChemiDoc MP System (Bio-Rad), and bands were quantified using Image Lab™ software (Bio-Rad). The expression of proteins was normalized to protein levels of a housekeeping protein (β-actin or Gapdh), and data are expressed as arbitrary units.

### Cytotoxicity assays

To determine the cytotoxic effect of compounds on MCECs, MCECs were seeded in MCEC growth medium without FBS supplemented at a density of 15,000 cells per well in wells of a 96-well plate that were pre-coated with 0.02% gelatin. Cells were treated with the given compounds at the given concentrations for 24 h, and cytotoxicity was measured using an LDH-Cytotoxicity Calorimetric Assay Kit (BioVision) according to the manufacturer’s instructions.

### Adipocyte area and multilocularity

AT samples were fixed in 4% paraformaldehyde overnight at 4 °C and then transferred to PBS in which they were stored at 4 °C until immunohistochemical analysis. Fixed AT was embedded in paraffin and sectioned into 5 μm sections. Adipocyte area and proportion of adipocyte population showing multilocularity were determined using Cell P (Olympus) and ImageJ software from hematoxylin and eosin (H&E)-stained sections imaged using an Olympus BX41 light microscope coupled to a ColorView digital color camera. At least three sections per tissue per animal were quantified blind.

### Flow cytometry staining and analysis

ScW from 12-week-old *Bmp8b* WT and TG mice was dissociated by collagenase treatment isolating unilocular adipocytes from the stromavascular fraction (SVF). The SVF was then kept in FACS buffer (PBS, 1 mM EDTA, 3% HI-FBS) on ice. Cells were stained with LIVE/DEAD (Invitrogen) and non-specific binding was blocked with 5 μg/ml anti-CD16/32 (Clone 93, Biolegend). Cell surfaces were then stained with anti-CD45 (30-F11, BD Biosciences), anti-CD31 (MEC 13.3, BD Biosciences), anti-CD34 (RAM 34, BD Biosciences) and anti-PDGFRα/CD140a (APA5, BD Biosciences). Cells were gated within the live cell population as CD45^–^, CD31^–^, CD34^+^ and PDGFRa^+^. Data were acquired on a Fortessa LSRII (BD Biosciences) using FACS Diva software and analyzed with TreeStar FlowJo.

### Magnetic-activated cell sorting

ScW from 12-week-old *Bmp8b* WT and TG mice was dissociated by collagenase treatment isolating unilocular adipocytes from the SVF. SVF was resuspended in MACS buffer (PBS, 2 mM EDTA (sterile), 0.5% bovine serum albumin) and sequentially incubated with Microbeads conjugated to monoclonal anti-mouse CD45 antibodies (Ly-5; isotype: rat IgG2b; clone:30-F11.1, Miltenyi Biotech), monoclonal antihuman/mouse CD11b (Mac-1α) antibodies (isotype: rat IgG2b, Miltenyi Biotech) and anti-CD31 antibody (isotype: ratIgG2a, Miltenyi Biotech) or 15 min at 4 °C. Finally, the CD45^+^, CD11b^+^ and CD31^+^ fractions were isolated using MACS LS columns according to the manufacturer's instructions (Miltenyi Biotech).

### Sympathetic nerve recordings

Sympathetic nerve activity (SNA) was measured directly from nerves supplying interscapular BAT and WAT as previously described^17^. Baseline SNA and hemodynamic variables were recorded for 10 min with rectal temperature maintained at 37.5 °C. An average of three separate measurements during the control period was considered to be the baseline value. After baseline BAT SNA was taken, using ice packs the rectal temperature of each mouse was lowered at constant and controlled rate, which was the same for each animal (0.25 °C/min). SNA to BAT was measured every 2 min. Background noise was subtracted from total integrated voltage to calculate real SNA to each tissue. Sympathetic nerve responses are expressed as percentage change from baseline.

### Phospho-antibody array

Brown differentiated adipocytes were treated with human recombinant BMP8 (100 pM) or NE (75 nM) or the combination of both prior to phosphoproteome assay. Cell lysates were applied to the Phospho Explorer Antibody Array (PEX100), which has been designed and manufactured by Full Moon Biosystems Inc. as previously published^49,50^. We verified the sequence position of phosphosites in UniProt sequences using data from PhosphoSitePlus^51^ (see Supplementary information). We looked up the potential kinases for the phosphosites in dbPTM^51^, HPRD^52^, Li 2012^53^, MIMP^54^, phospho.ELM^55^, PhosphoNetworks^56^, PhosphoSitePlus and Signor^57^ databases. We also considered the regulatory effects of the phosphosites, i.e., whether the phosphorylation is known to induce or disrupt the substrate’s interaction with other proteins, using the regulatory sites dataset from PhosphoSitePlus^51^. For the processing of the above-mentioned databases we used the pypath Python module that accompanies Omnipath^58^. We selected GO Biological Process terms^59^ to define five functions we were particularly interested in (angiogenesis; cell cycle and survival; inflammation; lipid metabolism and thermogenic activity; and neurogenesis) and we determined if the proteins showing high fold change on the phosphoassay, or their kinases or the downstream proteins regulated by the phosphorylation events, are annotated with any of these GO terms. To have a more unbiased insight into the biological functions of the observed patterns, we selected the phosphosite with the highest absolute fold change for each protein, a pathway enrichment analysis using the “piano“ R package was also performed^36^.

### Network analysis

We built a directed signaling network comprising 4557 nodes and 17,297 edges using the pathway dataset of OmniPath (10.1038/nmeth.4077). For all proteins with expression measured by qPCR in BAT of *Bmp8b* TG mice we collected the TFs regulating their expression using data up to confidence level D from the TF Regulons database^60^. We calculated the distances in this network from each of the proteins measured on the phosphoassay to all TFs using random walks with return, performing 15 iterations with 0.5 return probability according to equation 1 in ref. ^61^. We estimated the TF activities based on the qPCR measured expression data from *bmp8b* TG vs. WT mice using the ”msviper” method from the ”viper” R package (10.1038/ng.3593). We tested whether the proteins with phosphosites altered upon BMP8 treatment (phosphorylation fold change greater than 1.5) are closer in the network to the TFs of the proteins differentially expressed in BAT of *Bmp8b* TG mice. We found that although their distance is significantly lower by Mann–Whitney test, the difference is very small (not shown). We selected the 30 signaling proteins from the phosphoassay with lowest distances to *nrg4* and *vegfa*, the neurogenic and angiogenic factors with the most altered expression, and we show the phosphorylation fold change of these in Fig. 8a, b. We selected the TFs of *vegfa* with the most altered activities in *bmp8b* TG vs WT mice (*p* value < 0.2), and we show the normalized enrichment scores of these in Fig. 8c. All scripts used for the analysis can be found in https://github.com/saezlab/bmp8b.

### Statistics

The number of animals or independent experiments were determined based on pilot data and are all indicated in the figure legends. No statistical methods were used to predetermine the total number of animals needed for this study. The experiments were not randomized. The investigators, while blinded during the experiments and assessment of the outcome, were not blinded to the animals’ allocation. Mice were divided in groups with the same average body weight per group. All data from experiments is represented as a mean, with error bars showing standard error of the mean and the number of replicates stated in the figure legends. Some data are represented as a fold change, and it is stated in the figure legends to what value the data represented was normalized to generate the fold change. Statistical analyses and Grubbs’ tests to exclude outliers were performed using Prism6 software (GraphPad). The tests used are also stated in the figure legends. A *t*-test with equal variances was applied to compare two groups; one-way analysis of variance (ANOVA) or repeated measures ANOVA were used to compare more than two groups, followed by Bonferroni’s or Sidak post-hoc test. When more than one factor influenced the variable being measured, two-way ANOVA was used to test for a significant effect of each factor as well as an interaction between factors followed by Sidak post-hoc test. Gene panels were analyzed using multiple *t-*tests with a FDR determined using the two-stage linear step-up procedure of Benjamini, Krieger and Yekutieli, with *Q* = 1%.

## Electronic supplementary material

Supplementary Information

Supplementary Data 1

Supplementary Data 2

Supplementary Data 3

Supplementary Data 4

## Data Availability

Data that support the findings of this study including the scripts used for the analysis of the phosphoproteomics array can be found in Supplementary software. All other relevant data are available from the corresponding author on reasonable request.
