# Supplementary material for: Adipocyte-secreted BMP8b mediates adrenergic-induced remodeling of the neuro-vascular network in adipose tissue
Source: Nat Commun. 2018 Nov 26;9:4974. doi: 10.1038/s41467-018-07453-x (PMC6255810; doi:10.1038/s41467-018-07453-x)
Supplement: Supplementary file 1 — Supplementary Information [file 41467_2018_7453_MOESM1_ESM.pdf]

Supplementary Information

Adipocyte-secreted BMP8b mediates adrenergic-induced remodeling of the neuro-vascular network in adipose tissue

V Pellegrinelli & VJ Peirce et.al

Correspondence: vp332@ medschl.cam.ac.uk (V.P); ajv22@medschl.cam.ac.uk (A.V.-P.).

Supplementary Information includes 12 Figures and 2 tables

SUPPLEMENTARY FIGURES

Supplementary Figure 1:

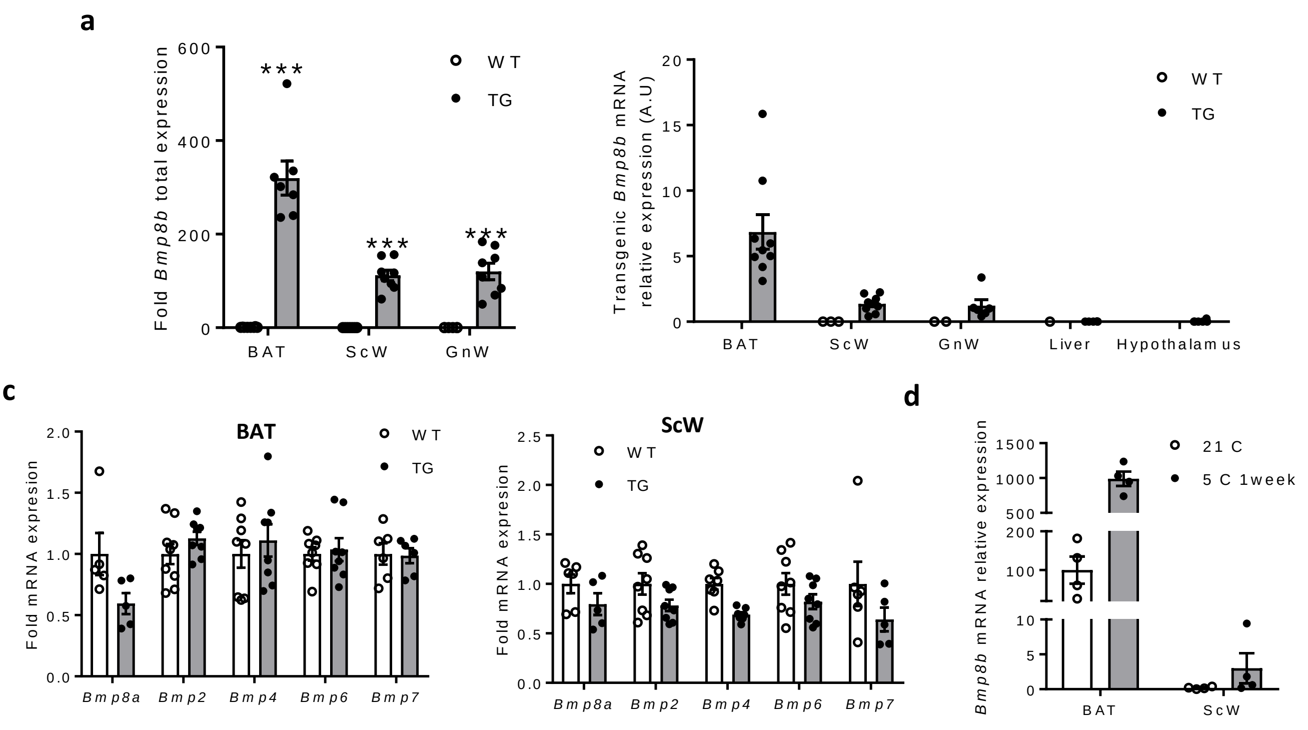

Supplementary Figure 1: BMP8b expression profile in TG and cold exposed mice

a, b:Total (a) and transgenic (b) *Bmp8b* mRNA levels in tissues from 5-week-old *Bmp8b* WT and TG mice, n = 5-9. * p < 0.05 compared to WT using student's t-test. n.d. non determined.

c: mRNA levels of *Bmp8B, Bmp2, Bmp4, Bmp6, and Bmp7* in interscapular BAT (b) and ScW (c) from 5-week-old WT and *Bmp8b* TG mice n =5-9. Data is represented normalized to the WT value. * p < 0.05 compared to WT using student's t-test, ns = not significant.

d: *Bmp8b* mRNA levels in interscapular BAT and inguinal ScW from 18-week-old WT male mice acclimated to 21°C and then exposed to 5 °C for 1 week, n = 4. Data is represented normalized to the BAT 21°C acclimated value (100). * p < 0.05 compared to 21 °C acclimated using Student's t-test.

*< 0.05, compared to wt using two way anova with sidak's post-hoc multiple comparisons test (a, d). *p<0.05, compared to wt using multiple t-test with fdr determined using the two-stage linear step-up procedure of Benjamini, Krieger and Yekutieli (c).

Supplementary Figure 2:

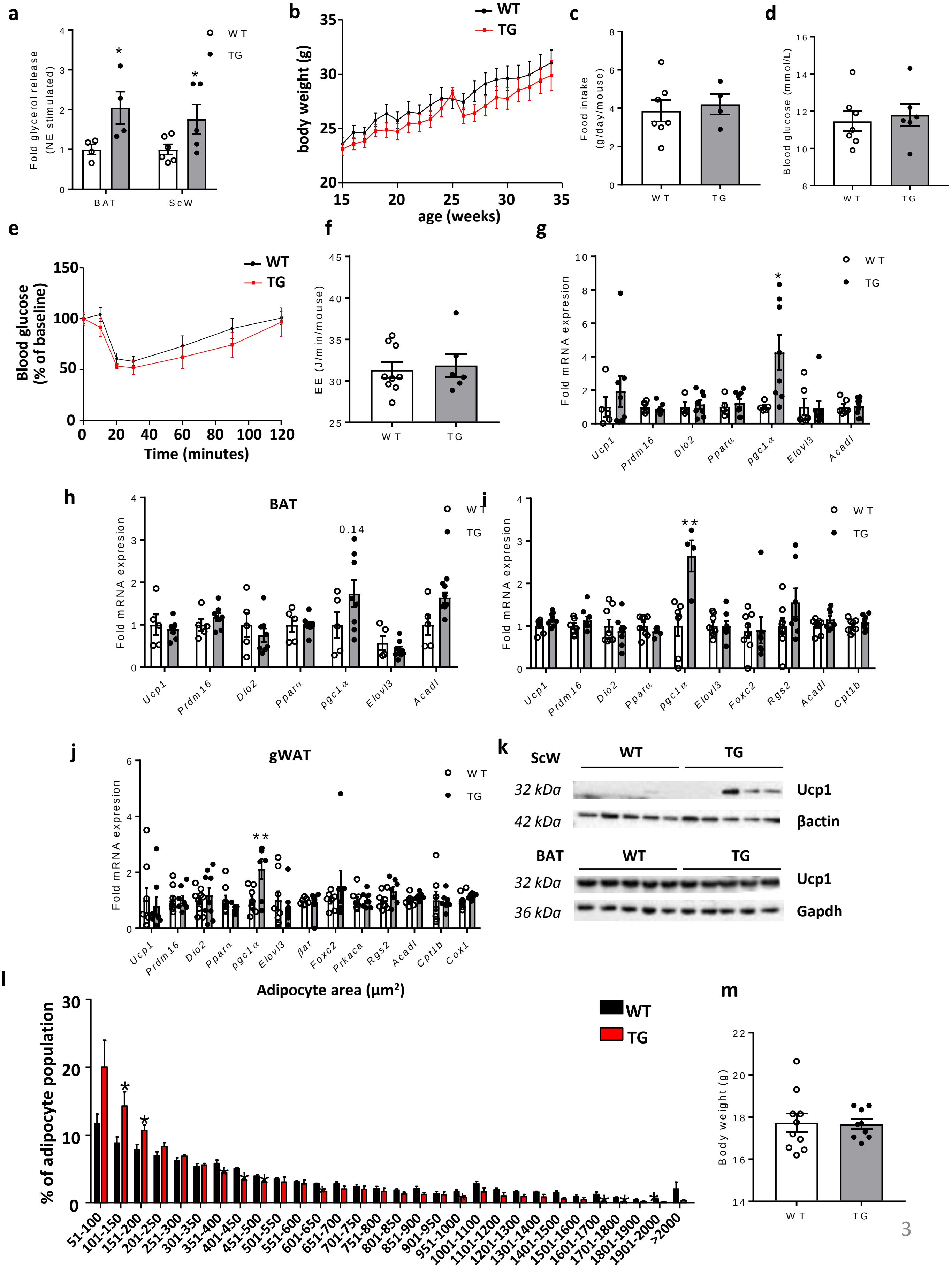

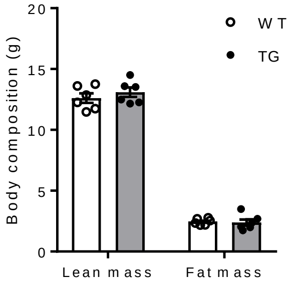

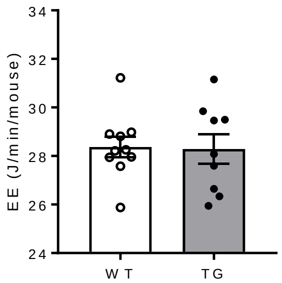

Supplementary Figure 2: BMP8b overexpression on metabolism and BAT/ScW expression profile

a: NE (100nM) stimulated lipolysis in mature brown or white adipocytes isolated from BAT and ScW of 10 weeks-old female wild type (WT) and *Bmp8b* TG (TG) mice housed at 21°C. Results are expressed in fold glycerol release over WT. n=4-6

b, c: Body weights (b) and food consumption (c) from 15-35 weeks old *Bmp8b* TG and WT mice (chow fed, housed at 21°C, n = 4-8.

d, e: Fasting blood glucose levels (d) and blood glucose levels represented as percentage of baseline up to 120 minutes after an intraperitoneal injection of a fixed dose of insulin (0.75 IU/kg; B) in an insulin tolerance test (ITT) (e) in 6-month-old WT and *Bmp8b* TG female mice fed a 45% HFD for 4 months, n = 6-7.

f: Energy expenditure (EE) of 6-month-old WT and *Bmp8b* TG mice (n =6-9) fed a 45% HFD for 4 months

g-i: Relative mRNA levels of thermogenic genes in ScW(g) and interscapular BAT (h, i) from 8-month-old (g, h) and 5-weeks-old (i) *bmp8b* WT and TG mice, n = 5-8. Data is represented normalized to the WT value.

j: Relative mRNA levels of thermogenic genes in GnW from 5-weeks-old *Bmpb* WT and TG female, n = 7-9. Data is represented normalized to the WT value.

k: UCP1 protein levels in BAT and ScW from 5-week-old *Bmp8b* TG and WT mice. n = 5

l: Quantification of the adipocyte area in inguinal ScW from 5-week-old WT and *Bmp8b* TG female mice. n = 5.

m-o: Body weight (m) body composition (n) and EE (o) from 5-week-old *Bmp8b* TG and WT mice of 5-week-old WT and BMP8bTG (TG) mice, n=6-10.

* $p < 0.05$ compared to WT using student's t-test (a, l). *$p<0.05$, compared to WT using multiple t-test with FDR determined using the two-stage linear step-up procedure of Benjamini, Krieger and Yekutieli (g, j).

Supplementary Figure 3:

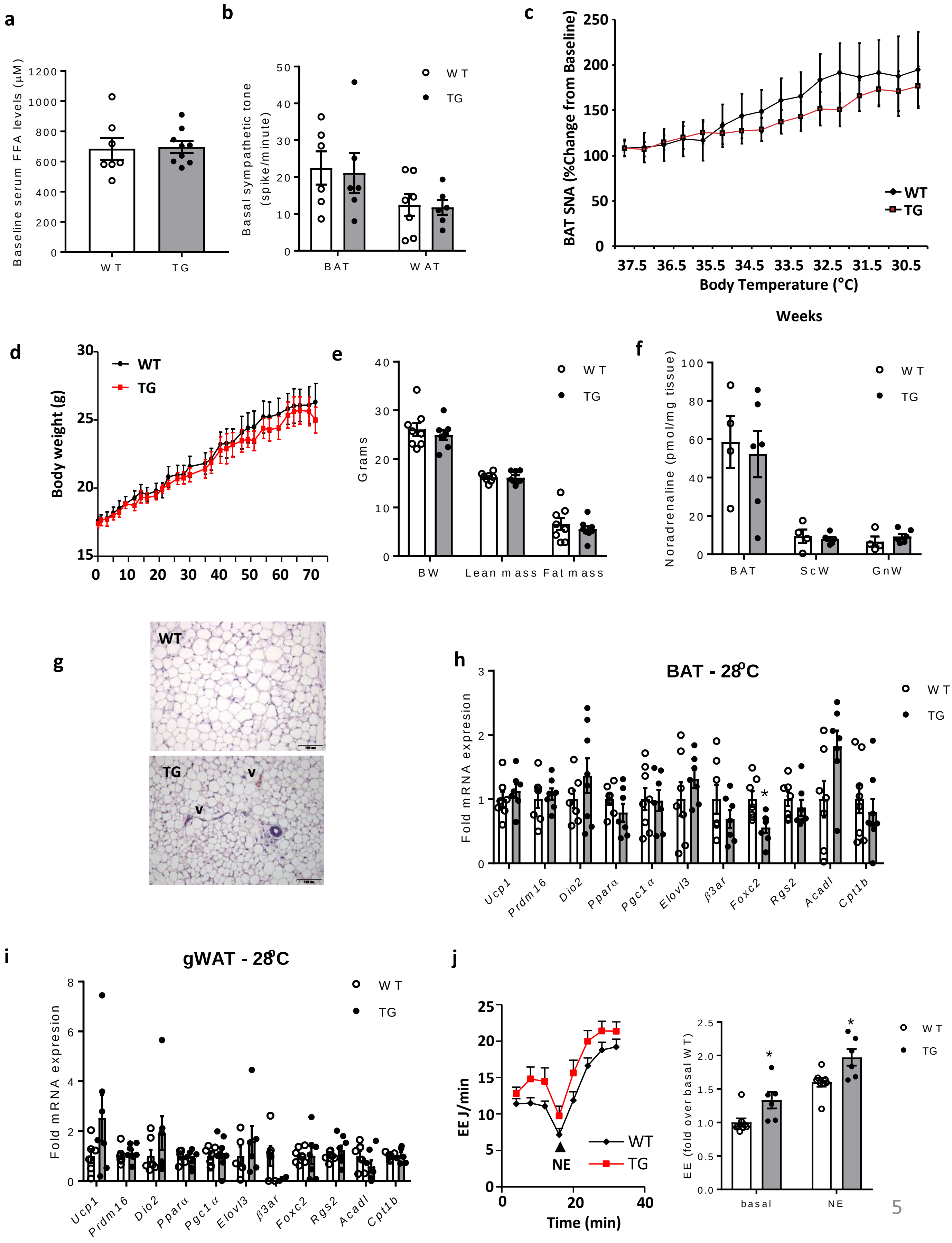

Supplementary Figure 3: Effect of thermoneutrality in BMP8b TG mice

a: Baseline FFA levels in the serum of 12 weeks-old *bmp8b* WT anf TG mice. n=7-9.

b, c: Direct measurement of SNA to BAT and WAT of 12 weeks old bmp8b WT and TG mice in baseline (b) and in response to controlled lowering of body temperature for 30 min (c). n =6-7

d, e: Body weights (d) and body composition (e) from *Bmp8b* TG and WT female mice over the course of 10 weeks of HFD-feeding at 28 °C. n = 8

f: Noradrenaline measurement in adipose tissue from 5weeks-old *bmp8b* WT and TG animals born and housed at 28C. n= 4-6.

g: Representative images of haematoxylin and eosin (H&E) staining in inguinal ScW from 5-week-old WT and TG female mice born and housed at 28 °C. n = 5.v, vessels.

h,i: Fold variation of mRNA levels of molecules that regulate thermogenesis in BAT (h) and GnW (i) from 5-week-old *Bmp8b* WT and TG mice born and housed at 28°C. n = 7-8. * p< 0.05 compared to WT using Mann Whitney test

j: NE stimulated Energy Expenditure (EE) in 12 weeks old WT and *Bmp8b* TG (TG) mice born and housed at 28°C, expressed as EE over time (genotype effect p<0.0001) and fold increase over basal condition, n = 6-8 *< 0.05 one-way ANOVA with Bonferroni post-hoc test

*p<0.05, compared to WT using multiple t-test with FDR determined using the two-stage linear step-up procedure of Benjamini, Krieger and Yekutieli (h). *< 0.05 compared to WT using repeated measures ANOVA with Sidak's post-hoc multiple comparisons test (j).

Supplementary Figure 4

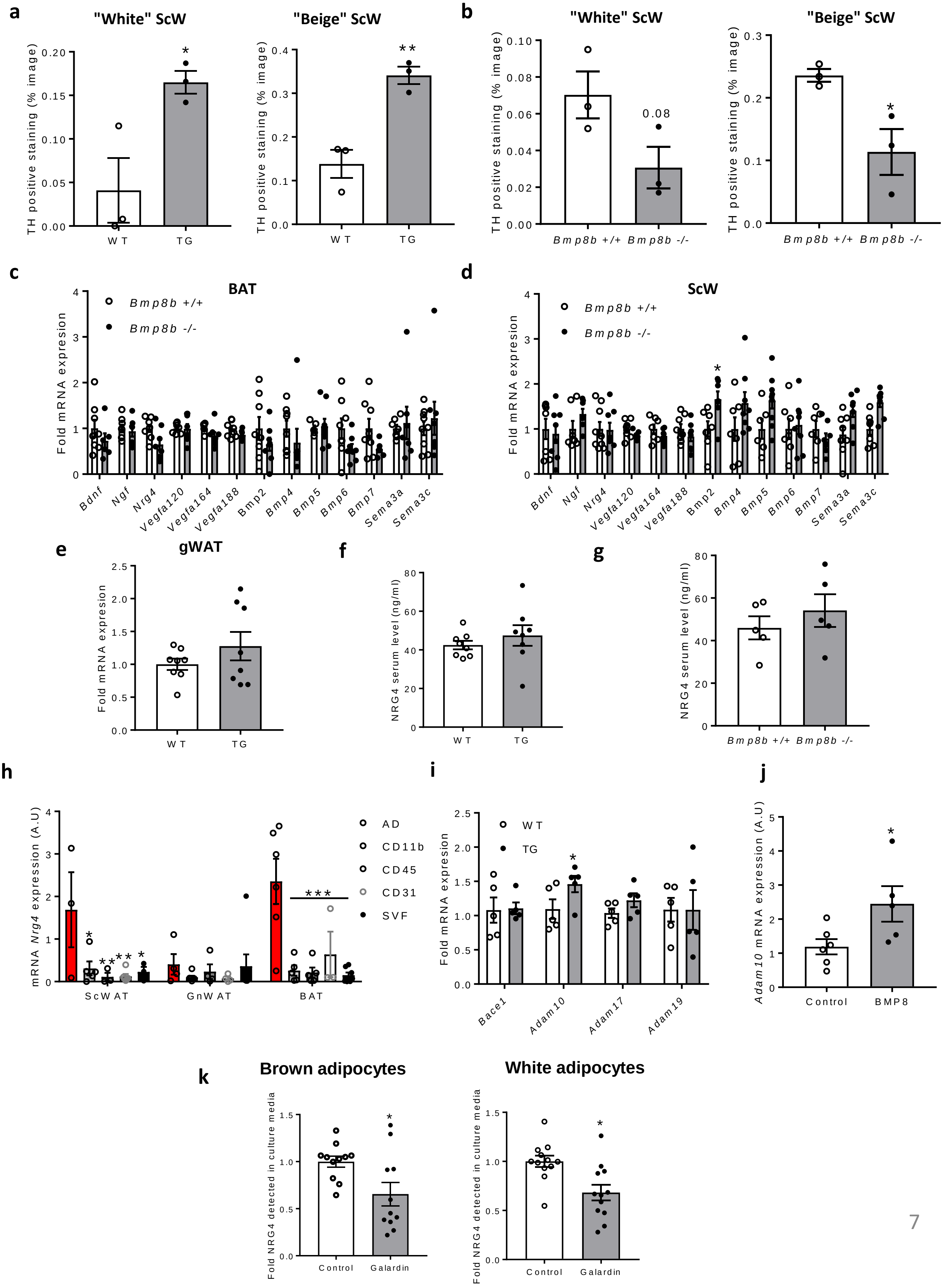

Supplementary Figure 4: BAT and ScW innervation in *Bmp8b* TG and -/- mice

a, b: Quantification of TH-positive staining in areas of purely beige adipocytes and areas of purely white adipocytes in ScW from 5-week-old female WT/*Bmp8b* TG (a) and *Bmp8b*$^{+/+}$/*Bmp8b*$^{-/-}$ (b) mice, n = 3.

c, d: Fold variation of mRNA levels of molecules that regulate sympathetic neuron survival, axonal or dendritic guidance or growth in BAT (c) and ScW (d) from 5-week-old WT and KO mice. n = 6-9.

e: Fold variation of *Nrg4* mRNA levels in GnW from 5-week-old WT mice. n = 8.

f, g: ELISA analysis of NRG4 levels in serum from WT vs *Bmp8b* TG (f), and *Bmp8b*$^{+/+}$/*Bmp8b*$^{-/-}$ (g), n=5-8.

h: Cell distribution of NRG4 expression in inguinal ScW , gonadal GnW and interscapular BAT from 10 weeks old WT mice (n=3-7). AD, adipocytes, CD11b, macrophages, CD45, immune cells and progenitors, CD31, endothelial cells and SVF^{-}, negative stroma-vascular fraction.

i, j: Expression of MMPs in primary brown adipocytes from *Bmp8b* WT and TG mice (i) and differentiated CS57 brown adipocytes treated during differentiation (9 days) with recombinant BMP8 (100pM) (j). n= 5-6. Data is represented normalized to the WT value.

k: Nrg4 secretion into the media by primary brown and white adipocytes treated with the broad-spectrum protease inhibitor galardin (10 µM for 24 hours). n= 12. Data is represented normalized to the WT value.

* p< 0.05 compared to WT using student's t-test (a, b, j, k). *p<0.05, compared to WT using multiple t-test with FDR determined using the two-stage linear step-up procedure of Benjamini, Krieger and Yekutieli (c, d, i). *p<0.05 compared to adipocytes (AD) using One-way ANOVA with Sidak's post-hoc multiple comparisons test (h).

Supplementary Figure 5:

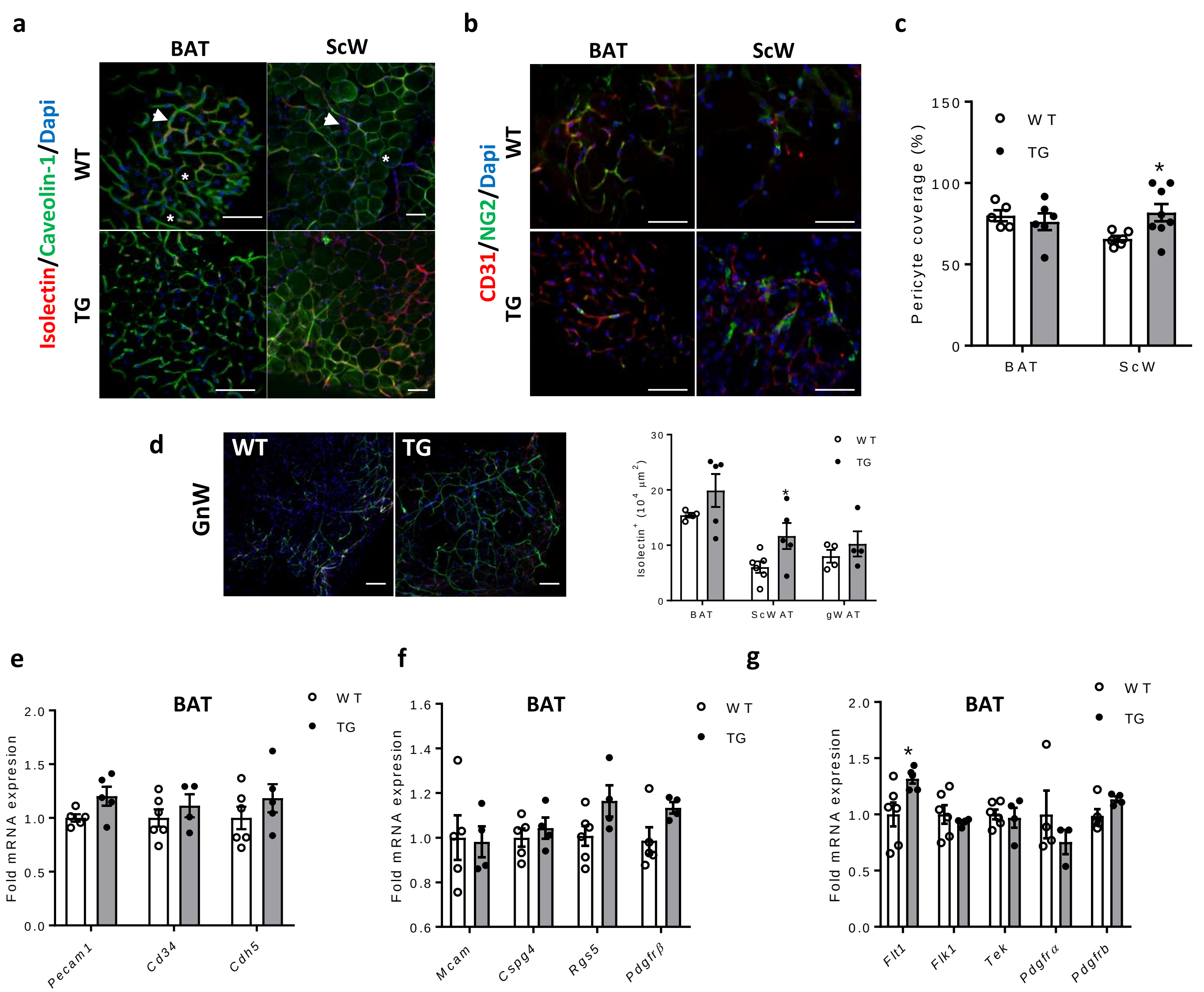

Supplementary Figure 5: Pericyte coverage in BAT/ScW and angiogenic profile in BAT from *Bmp8b* TG mice.
a: Representative images of confocal analysis of Isolectin IB4 and anti-caveolin-1 staining in BAT and ScW from 5-week-old WT and *Bmp8b* TG mice ; adipocytes are indicated with a white star and capillaries are tubular structures indicated by white arrows). Scale bar, 100µm.

b, c: Representative images of confocal analysis of CD31 (red, endothelial cells) and NG2 (green, pericytes) immunostainings in interscapular BAT and inguinal ScW from 5-week-old WT and *Bmp8b* TG (TG) mice (b) and quantification of pericytes coverage as percentage of pericytes in contact with endothelial cells (c). n=5-8.

d:Representative images of confocal analysis of Isolectin IB4 staining in GnW from 5-week-old WT and *Bmp8b* TG mice, and quantification of isolectin positive areas. n=4-6. Scale bar, 100µm.

e-g: Fold variation of mRNA levels endothelial cell (e), pericytes markers (f) and receptor of angiogenic factors (g) in BAT from 4-week TG female mice, n = 5-6. Data are expressed in fold variation over control WT littermate.
*p<0.05 compared to WT using Two-way ANOVA with Sidak's post-hoc multiple comparisons test (c). * p< 0.05 compared to WT using student’s t-test (d). *p<0.05, compared to WT using multiple t-test with FDR determined using the two-stage linear step-up procedure of Benjamini, Krieger and Yekutieli (g)

Supplementary Figure 6:

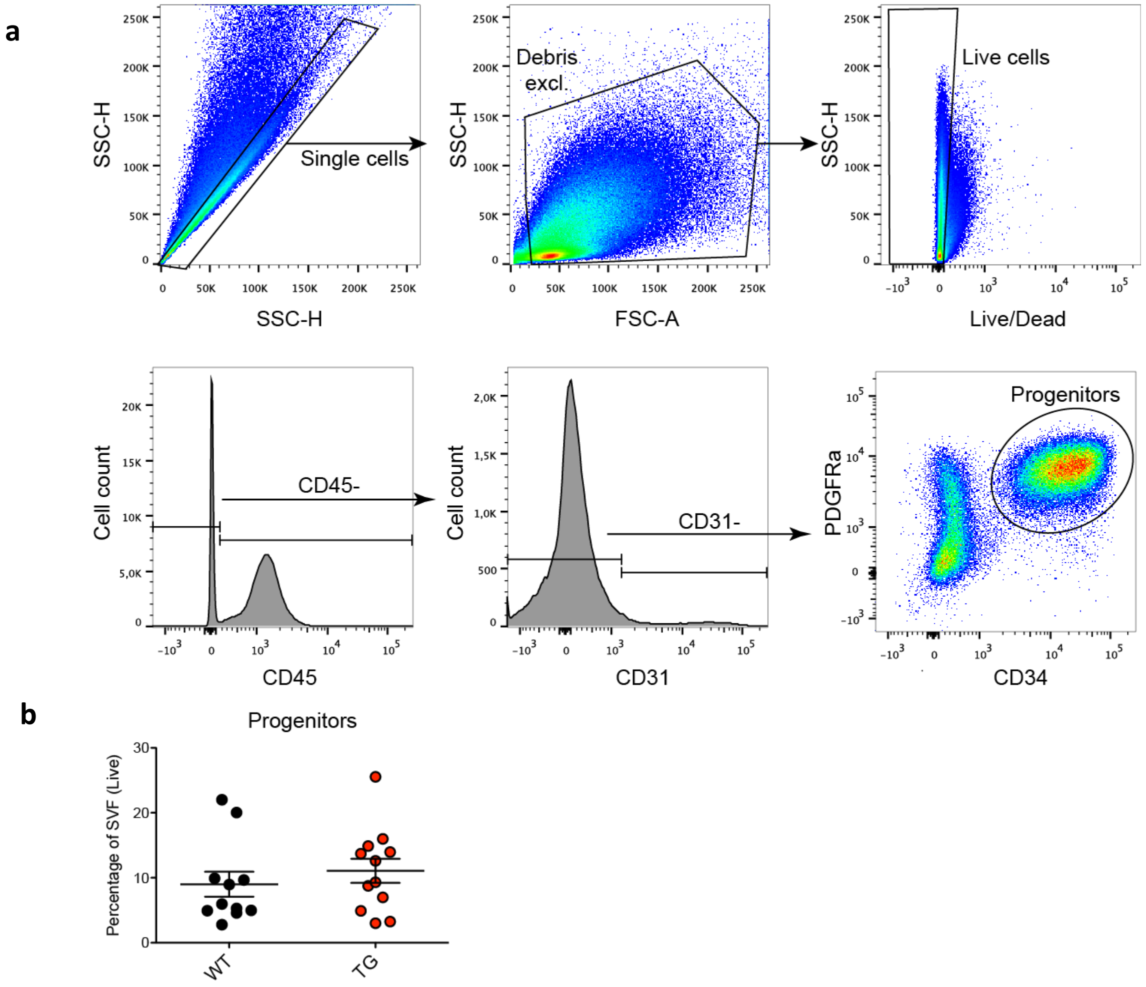

Supplementary Figure 6: Gating strategy for flow cytometry analysis of ScW progenitors.

a: SVF cells isolated from inguinal ScW of 12 weeks-month-old *Bmp8b* TG and WT mice were sequentially gated to discard doublets and clamps, debris and dead cells. The progenitors population ($CD34^+$ PDGFRα+) was gated on $CD45^-$ and $CD31^-$ cell populations.

b: Quantification of the number of progenitors expressed as percentage of the SVF population.

Supplementary Figure 7:

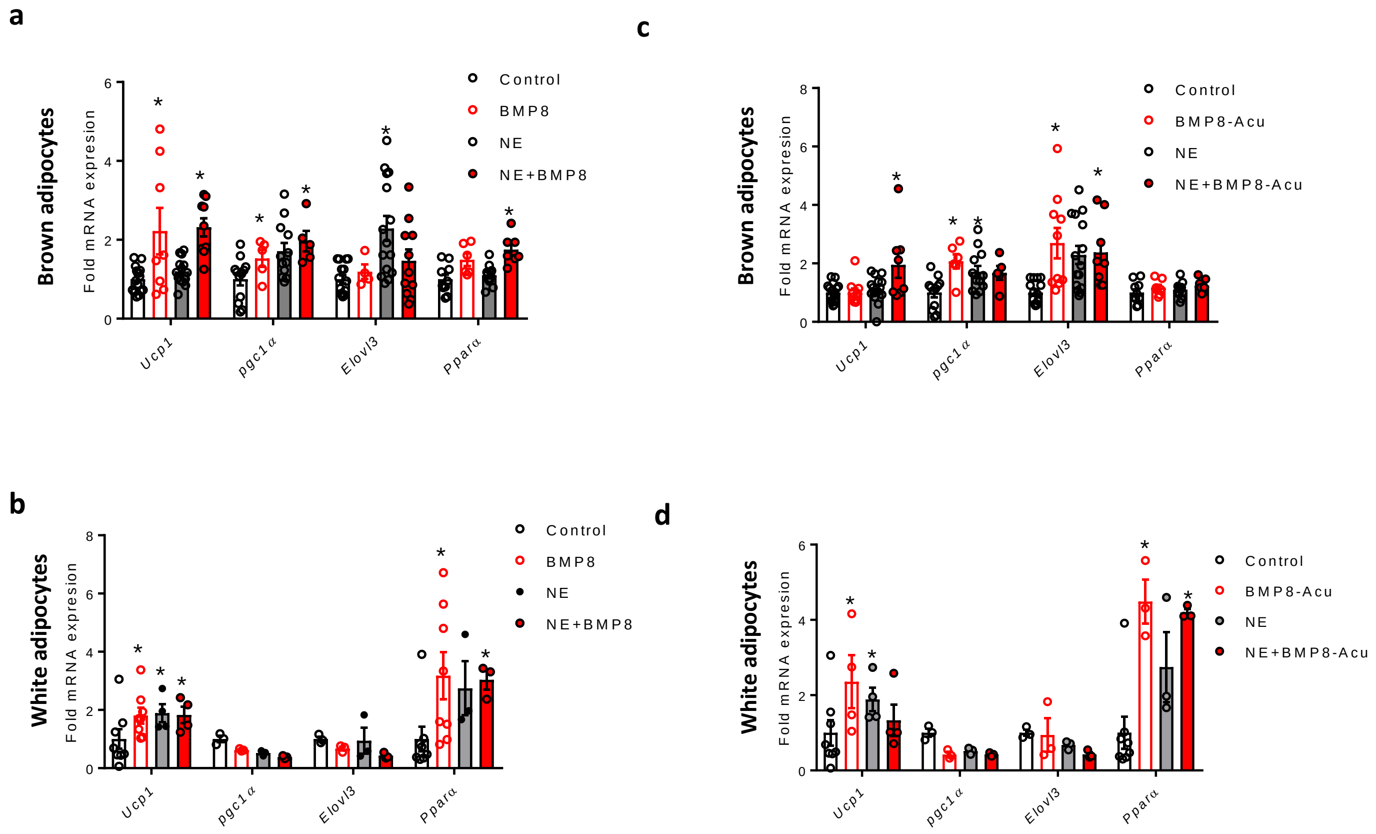

Supplementary Figure 7: Effect of BMP8 recombinant protein on thermogenic genes

a, b: Fold variation of relative mRNA levels of thermogenic genes in brown C57 adipocytes (a) and white 3T3L1 (b) differentiated adipocytes in control conditions or stimulated during the time of differentiation (9days) with recombinant human BMP8 protein (100pM, BMP8) and/or NE (75nM, BMP8 + NE and NE, respectively). n = 6-16.

c, d: Fold variation of relative mRNA levels of thermogenic genes in brown C57 adipocytes (c) and white 3T3L1 (d) differentiated adipocytes in control conditions or stimulated acutely (Acu) 24h with recombinant human BMP8 protein (BMP8-Acu) and/or NE (NE+BMP8b-Acu and NE, respectively). n = 3-8

*$p<0.05$ compared to control using One-way ANOVA with Sidak's post-hoc multiple comparisons test

Supplementary Figure 8:

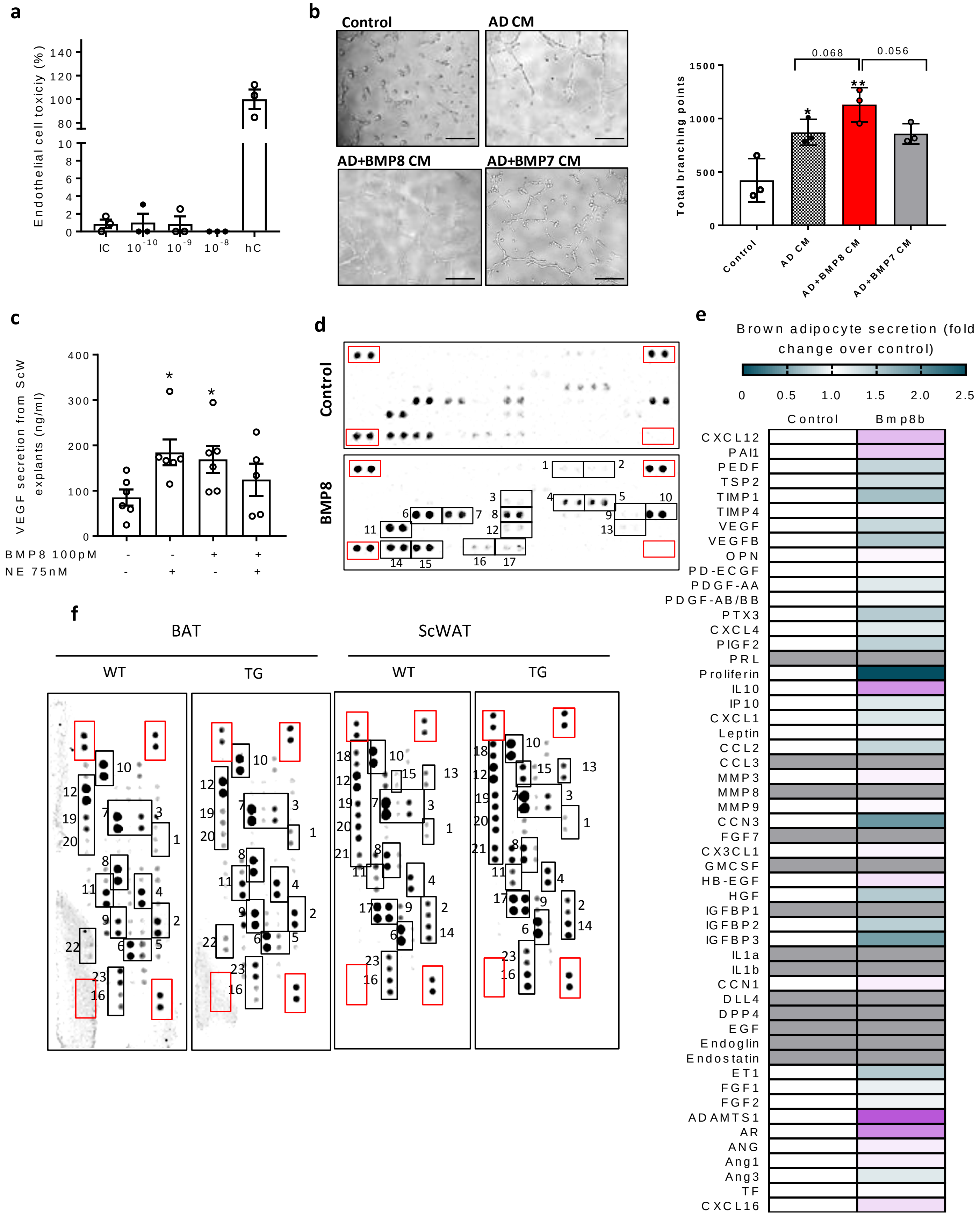

Supplementary Figure 8: Pro-angiogenic role of BMP8b

a: Effects of recombinant BMP8 on mouse cardiac endothelial cell (MCEC) toxicity (data representative of one experiment performed in triplicate). One-way ANOVA with Bonferroni post-hoc test comparing all data columns ($p < 0.05$).n=3.

b: Representative images and quantification of mouse cardiac endothelial cell (MCEC) vascular tube formation on Matrigel, without (control) or with conditioned medium (CM) prepared from brown adipocyte pre-treated without (AD CM) or with recombinant BMP8 (AD + BMP8 CM) or BMP7 (AD+BMP7 CM) (100pM, 24h). (n = 3 experiments in triplicate). Scale bar, 500µm.

c: ELISA analysis of VEGF secretion from ScW explants of 10 weeks old WT cultured in presence of NE or recombinant BMP8b, or both NE + BMP8b during 24h. n=6

d, e**:** Angiognesis Array of multiple analytes in culture medium of differentiated C57 brown adipocytes treated without (control) or with recombinant BMP8 (100pM, 30min). Each array was incubated with 1000 µL of a pool of 4 independent culture supernatant (d). Results are expressed in fold variation of protein levels of angiogenesis related factors and presented in a heat map with graded shades of magenta (down-regulated) and green (up-regulated) (e).
1:TF, 2:CXCL16, 3:HGF, 4:IGFBP-2, 5:IGFBP-3, 6:IP-10, 7:CXCL1, 8:CCL2, 9:MMP9, 10:CCN3, 11:OPN, 12:PTX3, 13:Proliferin, 14:CXCL2, 15:PAI-1, 16;TSP-2, 17: TIMP-1.

f: Angiognesis Array of multiple analytes in culture medium of BAT and ScW explants from 12 weeks-old *Bmp8b* TG and WT mice. Each array was incubated with 1000 µL of culture supernatant.
1:ANG,2: TF, 3:DPPIV, 4:Endostatin, 5:FGF-1, 6:IGFBP-3, 7:CXCL1, 8:CCL2, 9:MMP-3, 10:OPN, 11:CXCL4, 12:PAI-1, 13:ADAMTS, 14:CXCL16, 15:IP-10, 16:CCN1, 17:PlGF2, 18:CXCL12, 19:PEDG, 20TSP-2, 21:TIMP-1, 22:VEGF, 23:MMP9

*$p<0.05$ compared to IC (a) or control (b, c) using One-way ANOVA with Sidak's post-hoc multiple comparisons test (a, b, c)

Supplementary Figure 9:

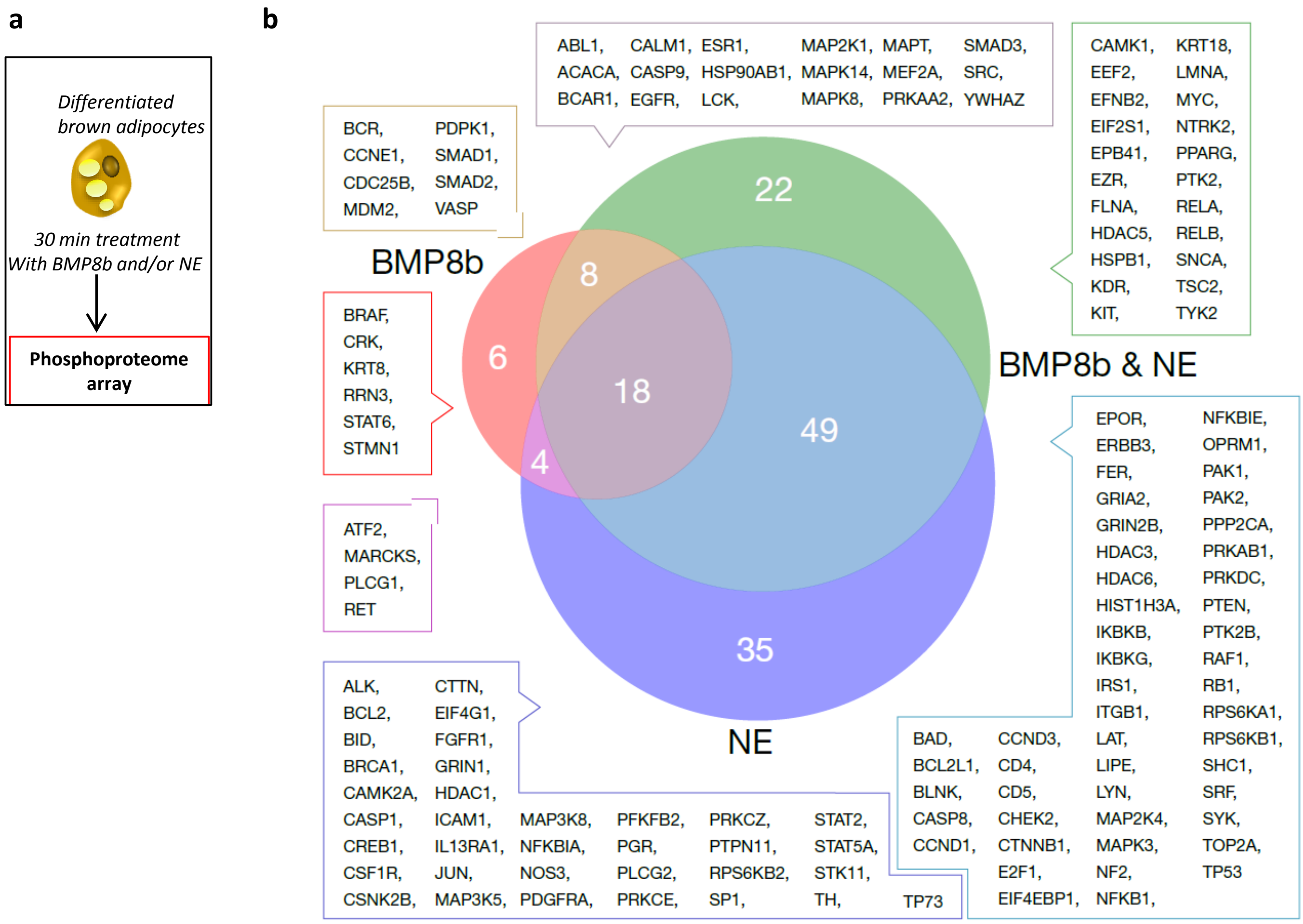

Supplementary Figure 9: Phosphoproteomic analysis in brown adipocytes treated with BMP8b±NE

a: Schematic of brown adipocyte treatment with or without (control) human recombinant BMP8b (100 pM) and/or NE (75 nM) prior phosphoproteome analysis

b: Venn-diagrams: Proteins with fold change values above 2 (twice more or half less phosphorylated) on any of their phosphosites between treatments. Proteins are represented by standard human gene names. The number of proteins in each field is proportional with the area of the field and shown by numbers.

Supplementary Figure 10:

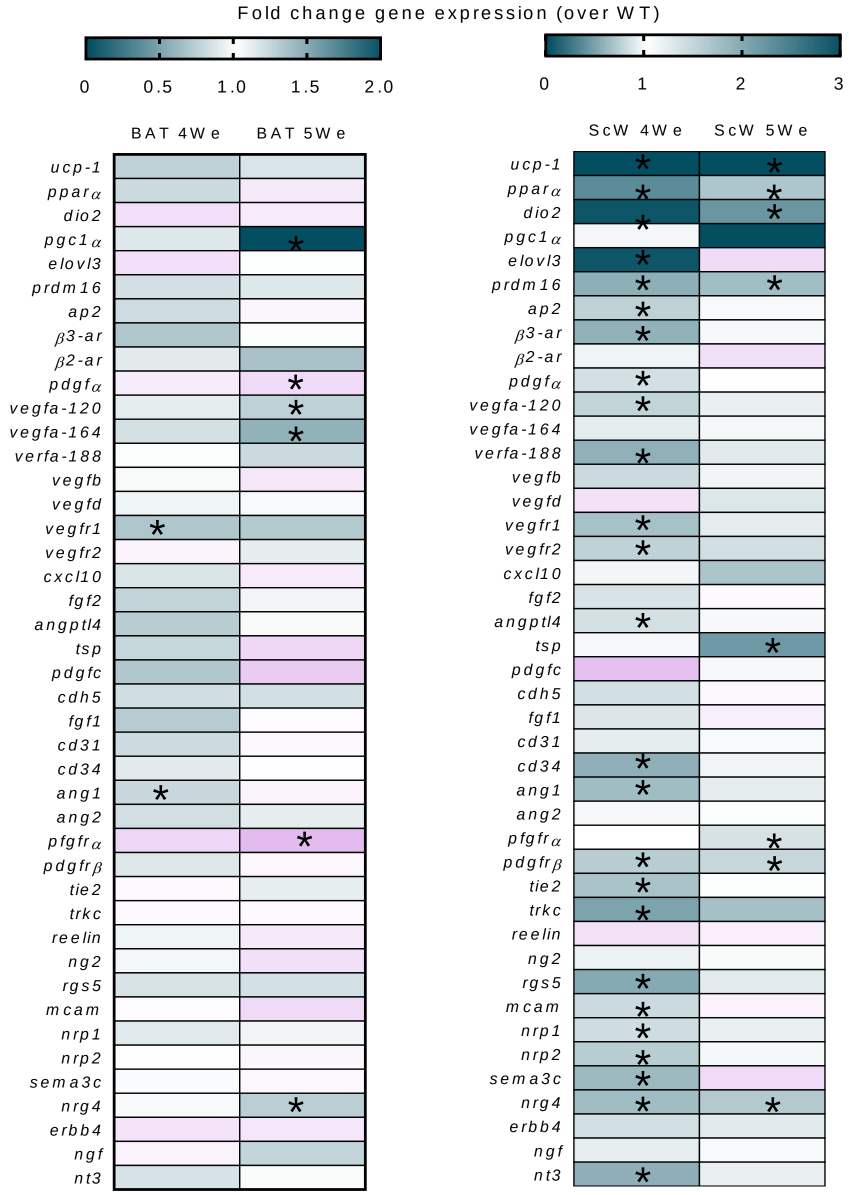

Supplementary Figure 10: Differential expression in 4 and 5 weeks old *Bmp8b* TG mice. Fold variation of mRNA levels of molecules that regulate thermogenesis, neurogenesis and angiogenesis in BAT and ScW from 4 and 5-week-old *Bmp8b* WT and TG mice. n = 6-8.

*p<0.05, compared to WT using multiple t-test with FDR determined using the two-stage linear step-up procedure of Benjamini, Krieger and Yekutieli .

Supplementary Figure 11:

a

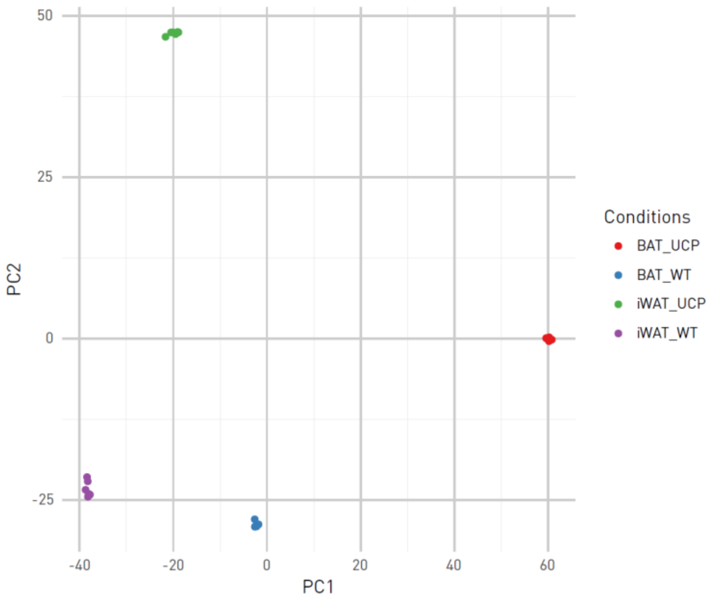

b

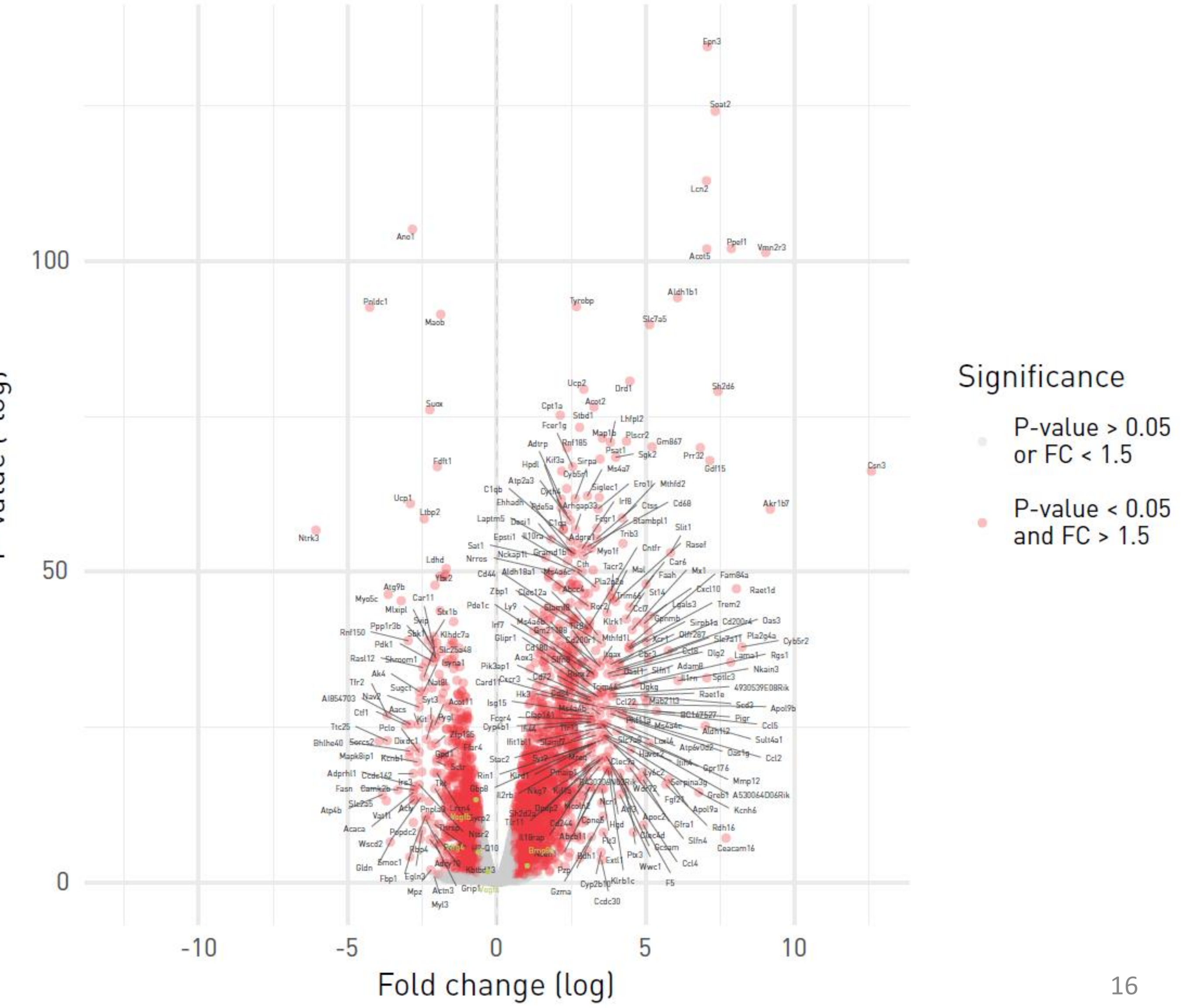

c

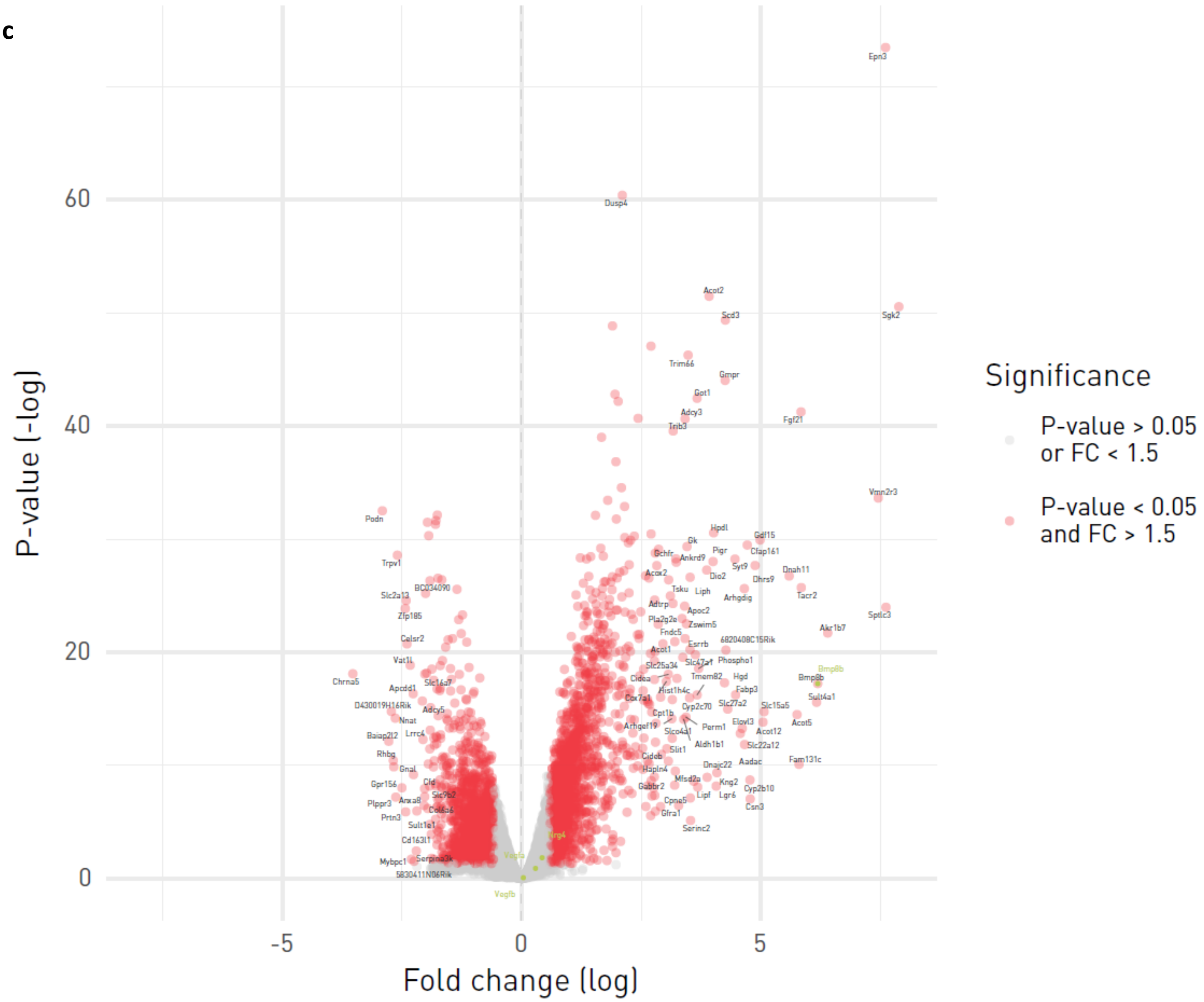

P-value (-log)
Fold change (log)
60
40
20
0
-5
0
5
Significance
P-value > 0.05
or FC < 1.5
P-value < 0.05
and FC > 1.5
Epn3
Dusp4
Acot2
Scd3
Sgk2
Trim66
Gmpr
Got1
Adcy3
Trib3
Fgf21
Vmn2r3
Podn
Trpv1
Slc2a13
BC034090
Zfp185
Calsr2
Vat1l
Chrna5
Apcdd1
Slc16a7
D430019H16Rik
Adcy5
Nnat
Baiap2l2
Lrrc4
Rhbg
Gnal
Gpr156
Cfd
Slc9b2
Plppr3
Anxa8
Col6a6
Prtn3
Sult1e1
Cd163l1
Mybpc1
Serpina3k
5830411N06Rik
Vegfb
Hpdl
Gk
Gdf15
Pigr
Cfap161
Ankrd9
Syt9
Dnah11
Acox2
Dio2
Dhrs9
Tsku
Liph
Arhgdig
Tacr2
Adtrp
Apoc2
Sptlc3
Pla2g2e
Zswim5
Akr1b7
Fndc5
Esrrb
6820408C15Rik
Acot1
Slc25a34
Slc47a1
Phospho1
Bmp8b
Cidea
Tmem82
Hgd
Hist1h4c
Fabp3
Sult4a1
Cox7a1
Cyp2c70
Slc27a2
Slc15a5
Cpt1b
Acot5
Arhgef19
Perm1
Elovl3
Slco4a1
Acot12
Aldh1b1
Slc22a12
Slit1
Cidec
Hapln4
Dnajc22
Aadac
Fam131c
Mfsd2a
Gabbr2
Kng2
Cyp2b10
Lipf
Lgr6
Cpne5
Csn3
Gfra1
Serinc2

d

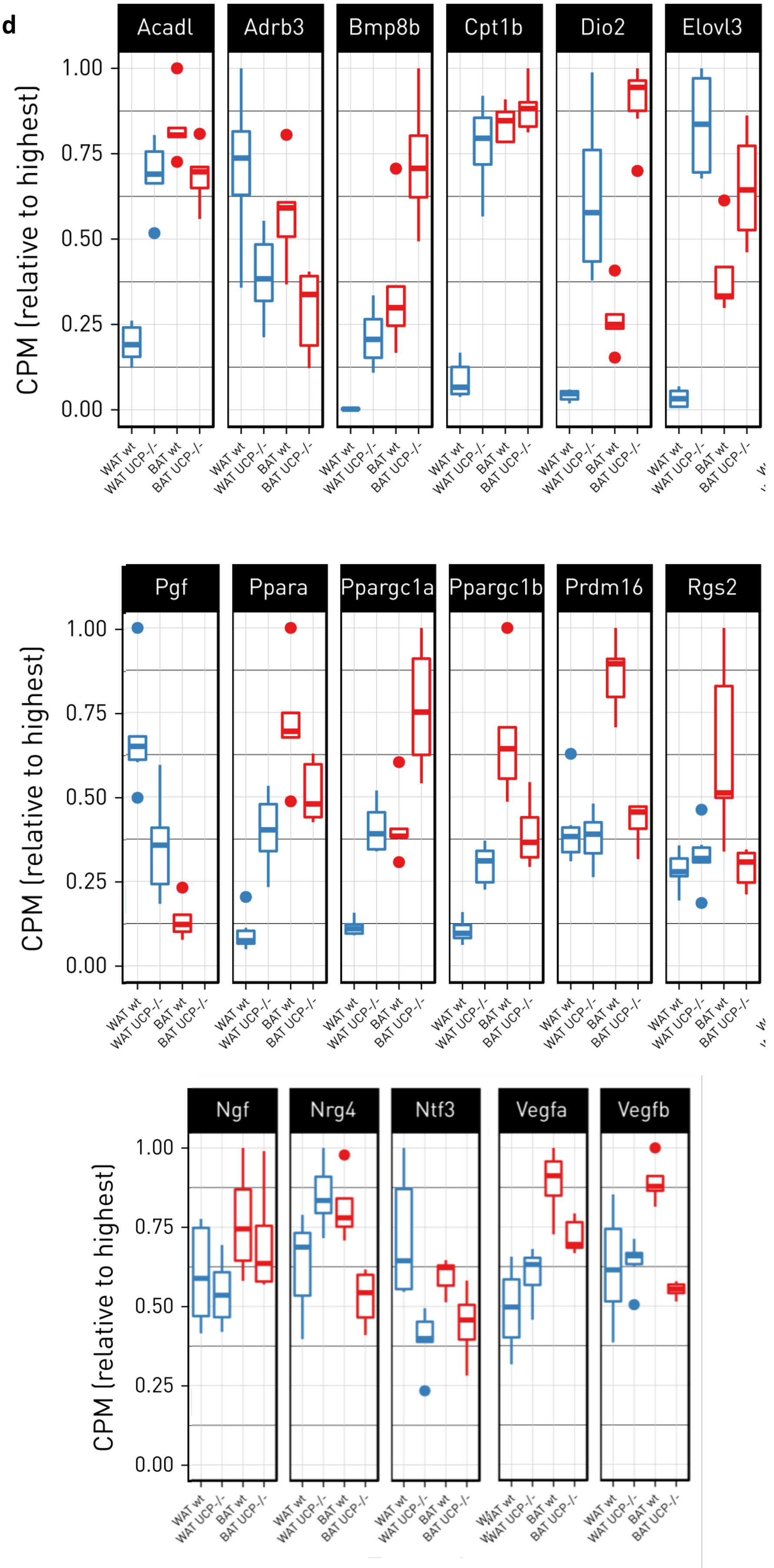

Acadl
Adrb3
Bmp8b
Cpt1b
Dio2
Elovl3
Pgf
Ppara
Ppargc1a
Ppargc1b
Prdm16
Rgs2
Ngf
Nrg4
Ntf3
Vegfa
Vegfb
CPM (relative to highest)
0.00
0.25
0.50
0.75
1.00
WAT wt
WAT UCP-/-
BAT wt
BAT UCP-/-

e

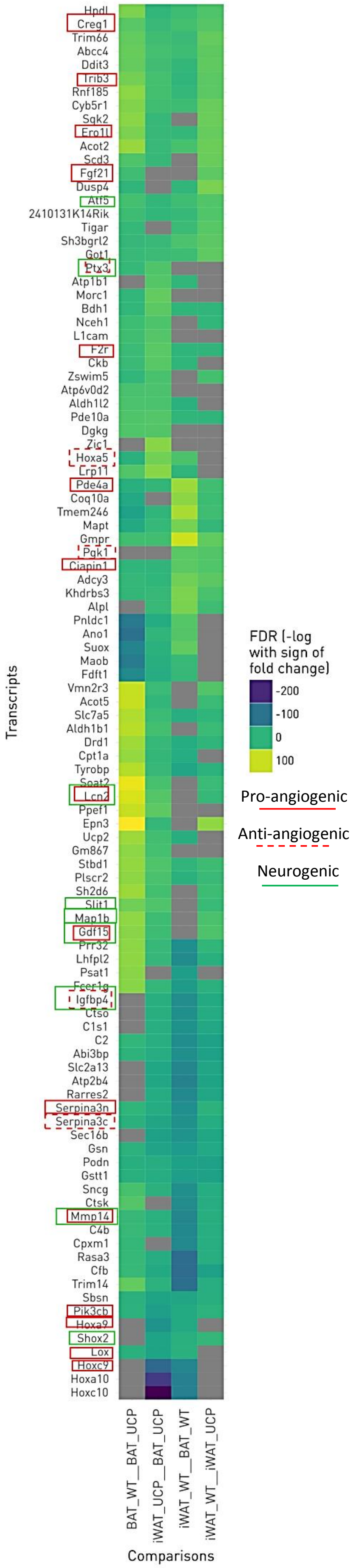

Hpdl
Creg1
Trim66
Abcc4
Ddit3
Trib3
Rnf185
Cyb5r1
Sgk2
Ero1l
Acot2
Scd3
Fgf21
Dusp4
Atf5
2410131K14Rik
Tigar
Sh3bgrl2
Got1
Ptx3
Atp1b1
Morc1
Bdh1
Nceh1
L1cam
F2r
Ckb
Zswim5
Atp6v0d2
Aldh1l2
Pde10a
Dgkg
Zic1
Hoxa5
Crp11
Pde4a
Coq10a
Tmem246
Mapt
Gmpr
Pgk1
Ciapin1
Adcy3
Khdrbs3
Alpl
Pnldc1
Ano1
Suox
Maob
Fdft1
Vmn2r3
Acot5
Slc7a5
Aldh1b1
Drd1
Cpt1a
Tyrobp
Soat2
Lcn2
Ppef1
Epn3
Ucp2
Gm867
Stbd1
Plscr2
Sh2d6
Slit1
Map1b
Gdf15
Prr32
Lhfpl2
Psat1
Fcer1g
Igfbp4
Ctso
C1s1
C2
Abi3bp
Slc2a13
Atp2b4
Rarres2
Serpina3n
Serpina3c
Sec16b
Gsn
Podn
Gstt1
Sncg
Ctsk
Mmp14
C4b
Cpxm1
Rasa3
Cfb
Trim14
Sbsn
Pik3cb
Hoxa9
Shox2
Lox
Hoxc9
Hoxa10
Hoxc10
Transcripts
BAT_WT__BAT_UCP
iWAT_UCP__BAT_UCP
iWAT_WT__BAT_WT
iWAT_WT__iWAT_UCP
Comparisons
FDR (-log with sign of fold change)
-200
-100
0
100
Pro-angiogenic
Anti-angiogenic
Neurogenic

Supplementary Figure 11: Transcriptomic analysis of *ucp-1 -/-* mice long term exposed to cold: Thermogenic and neuro-vascular gene expression profile. n=5-6

a: PCA of all genes demonstrating the distinct profile of *ucp-1* +/+ vs -/- and BAT vs ScW.

b, c: Volcano plot showing the distribution of genes (with high logFDR and high logFC) significantly (red) or not (grey) regulated in the BAT (b) and ScW (c) from *ucp-1* -/- mice compared to +/+ mice. *Bmp8b, nrg4,* and *vegfa/b* are presentedin yellow.

d: Box plots showing the transcript levels of thermogenic, neurogenic and angiogenic related genes.

e: The top 30 up- and downregulated unique transcripts between *ucp-1* +/+ and -/- mice were determined. The heatmap illustrates relative expression of the genotypes in BAT and ScW. Genes involved in angiogenesis and neurogenesis are boxed in red and green respectively.

Supplementary Figure 12:

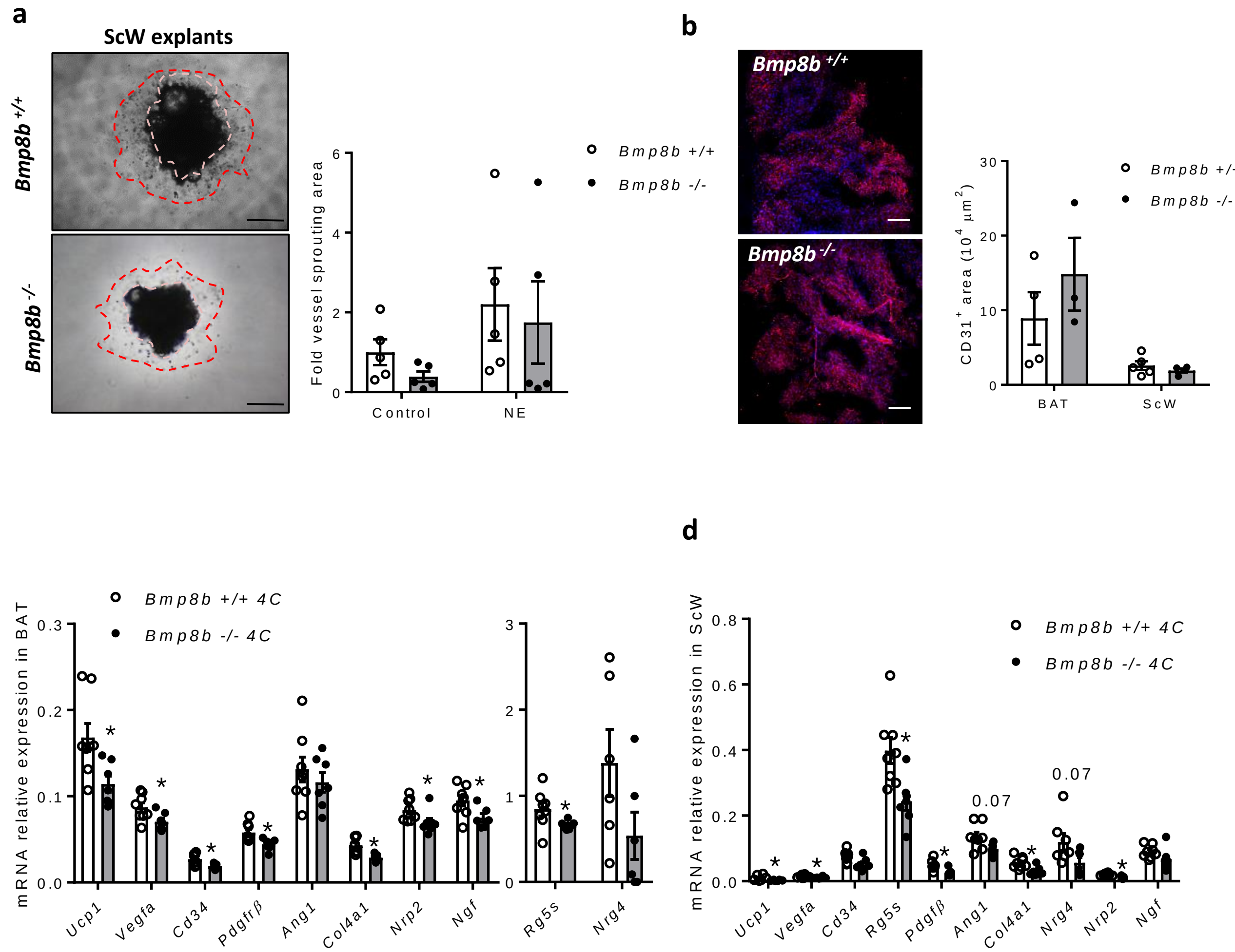

Supplementary Figure 12: Vascularization in *bmp8b* -/- mice

a: 10 weeks old WT and *Bmp8b*$^{-/-}$ mice ScW explants were cultured on Matrigel without (Control) or with NE-stimulation (10^{-7} M) for 10 days. Representative images of the cell sprouting in control conditions. Dotted white lines encompass the explant, dotted red lines show the cell sprouting area. Quantification of cell sprouting area, n = 5. Data is represented normalized to the WT control value. Scale bar, 500µm.

b: Representative images of confocal analysis of vascular features of inguinal ScW from 10-week-old WT and *Bmp8b*$^{-/-}$ (KO) mice : Vascular density (immunostaining for CD31 in red and quantification). n=3-5. Scale bar, 100µm.

c, d: Fold variation of mRNA levels of molecules that regulate neurogenesis and angiogenesis in BAT (c) and ScW (d) from 12-week-old *Bmp8b*$^{+/+}$ and *Bmp8b*$^{-/-}$ mice. n = 7-8. * p< 0.05 compared to WT using student's t-test.

Supplementary Table 1: Key Resources

REAGENT or RESOURCE	SOURCE	IDENTIFIER
**Antibodies**		
Rabbit polyclonal beta Actin (dilution 1/2000)	Abcam	ab8227
Mouse monoclonal [6C5] to GAPDH (dilution 1/1000)	Abcam	ab8245
Mouse monoclonal [LHM 2] to NG2 (dilution 1/1000)	Abcam	ab83508
Sheep polyclonal anti-Tyrosine Hydroxylase Antibody (dilution 1/200)	Chemicon Millipore	AB1542
Rabbit polyclonal anti-UCP1 antibody ((dilution 1/1000)	Abcam	ab10983
**Chemicals and Recombinant Proteins**		
Recombinant Human beta-NGF Protein	R&D systems	Mouse myeloma cell line, NS0-derived Ser122-Ala241
Caspase Inhibitor III	Calbiochem	CAS 634911-80-1
(±)-Norepinephrine (+)-bitartrate salt	Sigma	A0937-5G
Recombinant human BMP8a* (Bone morphogenic protein 8a)	R&D systems	1073-BP-010; E. coli-derived Ala264-His402 & Val265-His402
Recombinant human BMP7 (Bone morphogenic protein 7)	R&D systems	354-BP-010; Chinese Hamster Ovary cell line, CHO-derived human BMP-7 protein
Matrigel® Growth factor reduced Basement membrane Matrix	Corning	#354230
PuraMatrix® hydrogel	Corning Life Science	#354250
Recombinant human NRG4 (Neuregulin-4)	Life Technologies	12183HNCE25
ALK7 Inhibitor	Selleck Chemicals LLC	SB431542
**Critical Commercial Assays**		
ELISA kit for murine NRG4	Catlag Medsystems	SEC174Mu
ELISA kit for murine VEGF-A	R&D systems	DuoSet DY493-05
LDH-Cytotoxicity Calorimetric Assay Kit II	BioVision	#K313
**Experimental Models: Cell Lines**		
Immortalized brown adipocyte (C57BAT) cell line	Laboratory of Johannes Klein Supplementary reference 1	N/A
3T3L1 Cells	Zen-Bio	SPL1F
Mouse cardiac endothelial cell line (MCECs)	Cedarlane Laboratories	CLU510
**Experimental Models: Organisms/Strains**		
C57Bl/6J mice	Charles River	JAX™ Strain
Bmp8b -/- on a C57Bl6/J	Supplementary reference 2	N/A
Bmp8b TG mice, WT SV129*C57Bl/6J mixed strain mice and backcrossed until whole genome >98% C57Bl/6J genotype	In this paper	N/A
**Software and Algorithms**		
Zeiss LSM 510 Meta Confocal microscope with LSM 3D software	Carl Zeiss	N/A
Fiji software	ImageJ	N/A
Image Lab™ software	Bio-Rad	N/A
FACS Diva software	BD Biosciences	N/A
Prism 6	GraphPad	N/A
\`piano\` R package	Supplementary reference 3	N/A
pypath Python module that accompanies Omnipath	Supplementary reference 4	N/A
**Databases used for the phosphoproteomics analysis**		
PhosphoSitePlus	Supplementary reference 5	N/A
dbPTM	Supplementary reference 5	N/A
HPRD	Supplementary reference 6	N/A
Li 2012	Supplementary reference 7	N/A
MIMP	Supplementary reference 8	N/A
phospho.ELM	Supplementary reference 9	N/A
PhosphoNetworks	Supplementary reference 10	N/A
PhosphoSitePlus and Signor	Supplementary reference 11	N/A
TF Regulons database	Supplementary reference 12	N/A

*Recombinant human Bmp8a was used for Bmp8b treatment experiments on brown adipocytes, as in [17]. There is high protein sequence similarity between human Bmp8a, human Bmp8b, and murine Bmp8b, and previously the product was named human Bmp8b.

Supplementary Table 2-Primer sequences

Gene	Forward primer (5'-3')	Reverse primer (5'-3')	Probe (5'-3')	Reporter/quencher
18s	CGG CTA CCA CAT CCA AGG AA	GCT GGA ATT ACC GCG GCT	GAG GGC AAG TCT GGT GCC AG	FAM/TAMRA
36b4	AGA TGC AGC AGA TCC GCA T	GTT CTT GCC CAT CAG CAC C		SYBR
A1ar	AGA GAA AGC CGC CAA G	GGC TGG AGC ATG GGT ATA TG		SYBR
A2ar	CGC AGG CCA TCG AGT ACA A	GGT GAC AAT GAT GGC CTT GAT		SYBR
B3ar	CCA GCC AGC CCT GTT GA	GGA CGC GCA CCT TCA TAG C		SYBR
Bactin	GCT CTG GCT CCT AGC ACC AT	GCC ACC GAT CCA CAC AGA GT	ATC AAG ATC ATT GCT CCT GAG CGC	FAM/TAMRA
Acadl	GCA TGA AAC CAA ACG TCT GGA	TGT TTT GTA ATT CAG ATG CCC AGT	TCC GGT TCT GCT TCC ATG GCA AAA	FAM/TAMRA
Angpt1	TTT CTC TTC CCA GAA ACT TCA GC	GGC CAT CTC CGA CTT CAT ATT T		SYBR
Angpt2	CAG CAC AAA CTC GGA AAC TGA CT	AGC TTG TTT ATT TCA CTG GTC TGA TC		SYBR
B2m	ACT GAT ACA TAC GCC TGC AGA GTT	TCA CAT GTC TCG ATC CCA GTA GA		SYBR
Bdnf	GGG TCA CAG CGG CAG ATA AA	GCC TTT GGA TAC CGG GAC TT		SYBR
Bmp2	AAC ACC GTG CGC AGC TT	GCC GTT TTC CCA CTC ATC TC		SYBR
Bmp4	GCC ATT CCG TAG TGC CAT T	TTG CTG GAA AGG CTC AGA GA		SYBR
Bmp5	CAA GAA GCA CGA ACT CTA TGT GAG T	CCC TTC TGG TGC TAT GAT CCA		SYBR
Bmp6	CGC AGG ACA GCG CTT TC	GTC GTA CTC CAC CAG GTT CAC A		SYBR
Bmp7	TGG ATG GGC AGA GCA TCA A	GGT CCA TGC CGT CCA ATC		SYBR
Bmp8b endogenous	TCC ACC AAC CAC GCC ACT AT	CAG TAG GCA CAC AGC ACA CCT	CAG GCC CTG GTA CAT CTG ATG AAG CC	FAM/TAMRA
Bmp8b total	TCC ACC AAC CAC GCC ACT AT	CAG TAG GCA CAC AGC ACA CCT		SYBR
Bmp8b transgenic	TCC ACC AAC CAC GCC ACT AT	CAG TAG GCA CAC AGC ACA CCT	AGG CCC TGG TGC ACT TAA TGA	FAM/TAMRA
Bmp8a	AAC CAT GCC ATC TTG CAG TCT	CAG AGG TGG CAC TCA GTT TGG	TGT CCC CAA GGC ATG CTG TGC A	FAM/TAMRA
Cd34	CCC ACC GAG CCA TAT GCT T	ACC TCC TCA CAA CTA GAT GCT TCA CTT		SYBR
Cdh5	CTT CAA GCT GCC AGA AAA CC	GAA TTG GCC TCT GTC ACT GG		SYBR
Cpt1b	GCG TGC CAG CCA CAA TTC	TCC ATG CGG TAA TAT GCT TCA T		SYBR
Cspg4	AGC AAG GAA GTG CAG AGG AG	CAT CGA AAG ACA CCA TCA CG		SYBR
Cxcl10	CGT CAT TTT CTG CCT CAT CC	CGT GGC AAT GAT CTC AAC AC		SYBR
Cxcl12	TGC ATC AGT GAC GGT AAA CCA	TTC AGC CGT GCA ACA ATC		SYBR
Dio2	TGC GCT GTG TCT GGA ACA G	CTG GAA TTG GGA GCA TCT TCA		SYBR
Elovl3	AAG GTT GAA CTG GGA CGA C	GTG GTA CCA GTG GAC AAA		SYBR
F3	ACA ATT TTG GAG TGG CAA CC	TGG GAC AGA GAG GAC CTT TG		SYBR

Fgf1	**AGG GGA GAT CAC AAC CTT CG**	**CAC TTT CCG CAC TGA GCT G**		**SYBR**
Fgf2	ABI TaqMan Gene Expression Assay ID Mm00433287_m1			FAM/TAMRA
Flk1	GAT GCA GGA AAC TAC ACG GTC AT	AGG AAT CCA TAG GCG AGA TCA A		SYBR
Flk1	GAT GCA GGA AAC TAC ACG GTC AT	AGG AAT CCA TAG GCG AGA TCA A		SYBR
Foxc2	ABI TaqMan Gene Expression Assay ID Mm00546194_s1			FAM/TAMRA
Hgf	ATG TGG GGG ACC AAA CTT C	TCA GTA ATG GGT CTT CCT TGG		SYBR
Igf1	GAC CGA GGG GCT TTT ACT TC	GTC TTG GGC ATG TCA GTG TG		SYBR
Igf2	TCT CAT CTC TTT GGC CTT CG	GCA CTC TTC CAC GAT G		SYBR
Mcam	GTG CGT CTT GTT CGC TG	TGC CTC TCC TTG TGA ATC AAA AAC		SYBR
Ngf	AGT GTC TGG GCC CAA TAA AG	GTC TCC TTC TGG GAC ATT GC		FAM/TAMRA
Nrg4	ABI TaqMan Gene Expression Assay ID Mm00446254_m1			FAM/TAMRA
Pdgfa	CAC CAG CGT CAA G	GAT GGT CTG GGT TCA GGT TG		SYBR
Pdgfb	CCT TCC TCT CTG CTA CC	GTG TGC TCG GGT CAT GTT C		SYBR
Pdgfc	AGT TGA GGA GCC CAG TGA TG	CGA AGG ACT CGT GGT TTC TG		SYBR
Pdgfrα	AGC TGT CAA CTT GCA CGA AG	CCG GAT CAG CTT TAA TTT GC		SYBR
Pdgfrβ	CAT GTC TGA GAC CCG GTA CG	CTG CAG GTA GAC CAG GTG AC		SYBR
Pecam1	TCA GTC GGC AGA CAA GAT GCT	CCA TAT GGA TGC TGT TGA TGG T		SYBR
Pgc1α	AAC CAC ACC CAC AGG ATC AGA	CTC TTC GCT TTA TTG CTC CAT GA	CAA ACC CTG CCA TTG TTA AGA CCG AGA A	FAM/TAMRA
Pgf	TGT GTG CCG ATA AAG ACA GC	CCC TTG GTT TTC CTC CTT TC		SYBR
Pparα	GAT TCA GAA GAA GAA CCG GAA CA	GCG AAT TGC ATT GTG TGA CAT	TGC CGT TTT CAC AAG TGC CTG TCT GTC	FAM/TAMRA
Pparγ2	GAT GCA CTG CCT ATG AGC ACT T	AGA GGT CCA CAG AGC TGA TTC C	AGA GAT GCC ATT CTG GCC CCA CCA ACT T	FAM/TAMRA
Prdm16	CAG CAC GGT GAA GCC ATT	GCG TGC ATC CGC TTG TG		SYBR
Rgs2	GGA GAA AAT GAA GCG GAC AC	TTA CTG GCC AGC AGT TCA TC		SYBR
Rgs5	AGA GGC CCC TAA AGA GGT GA	AGT CAA AGC TGC GAG GAG AC		SYBR
Sema3a	GCT TTC CAT CCA ATC TGC AC	AGT CCC GTC CCA TGA AGT C		SYBR
Sema3c	GAG CAA AGA CCA CAT CCT GTC	GCT CCA CTC CCA CAG ACA TAC		SYBR
Tek	CTT CCA CAT TCC CCA GCC TC	ACT GCA CAG CTG GTT CTT CT		SYBR
Th	GAG AGC TTC AGT GAT GCC AAG	CAT CCT GGA CCC CCT CTA AG		SYBR
Thbs1	GAT GGG GTT GGA GAT CAG TG	TTG GCA TTA GGC ACA TAG GG		SYBR
Ucp1	CCC GCT GGA CAC TGC C	ACC TAA TGG TAC TGG AAG CCT GG	AAG TCC GCC TTC AGA TCC AAG GTG AAG	FAM/TAMRA
Vegfa120	GCC AGC ACA TAG AGA GAA TGA GC	CGG CTT GTC ACA TTT TTC TGG	ACA GCA GAT GTG AAT GCA GAC CAA AGA AAG	FAM/TAMRA
Vegfa164	GCC AGC ACA TAG AGA GAA TGA GC	CAA GGC TCA CAG TGA TTT TCT GG	ACA GCA GAT GTG AAT GCA GAC CAA AGA AAG	FAM/TAMRA
Vegfa188	GCC AGC ACA TAG AGA GAA TGA GC	AAC AAG GCT CAC AGT GAA CGC T	ACA GCA GAT GTG AAT GCA GAC CAA AGA AAG	FAM/TAMRA
Vegfb	CAC CCA GGC CCC TGT GT	CCC ATG AGT TCC ATG CTC AGA		SYBR

Supplementary References

1. Klein, S. *et al.* Alpha 5 beta 1 integrin activates an NF-kappa B-dependent program of gene expression important for angiogenesis and inflammation. *Mol. Cell. Biol.* **22,** 5912–5922 (2002).
2. Zhao, G. Q., Deng, K., Labosky, P. A., Liaw, L. & Hogan, B. L. The gene encoding bone morphogenetic protein 8B is required for the initiation and maintenance of spermatogenesis in the mouse. *Genes Dev.* **10,** 1657–1669 (1996).
3. nVäremo, L., Nielsen, J. & Nookaew, I. Enriching the gene set analysis of genome-wide data by incorporating directionality of gene expression and combining statistical hypotheses and methods. *Nucleic Acids Res.* **41,** 4378–4391 (2013).
4. Türei, D., Korcsmáros, T. & Saez-Rodriguez, J. OmniPath: guidelines and gateway for literature-curated signaling pathway resources. *Nat. Methods* **13,** 966–967 (2016).
5. Hornbeck, P. V. *et al.* PhosphoSitePlus, 2014: mutations, PTMs and recalibrations. *Nucleic Acids Res.* **43,** D512-520 (2015).
6. Keshava Prasad, T. S. *et al.* Human Protein Reference Database--2009 update. *Nucleic Acids Res.* **37,** D767-772 (2009).
7. Li, L. *et al.* The human phosphotyrosine signaling network: evolution and hotspots of hijacking in cancer. *Genome Res.* **22,** 1222–1230 (2012).
8. Wagih, O., Reimand, J. & Bader, G. D. MIMP: predicting the impact of mutations on kinase-substrate phosphorylation. *Nat. Methods* **12,** 531–533 (2015).
9. Dinkel, H. *et al.* Phospho.ELM: a database of phosphorylation sites--update 2011. *Nucleic Acids Res.* **39,** D261-267 (2011).
10. Hu, J. *et al.* PhosphoNetworks: a database for human phosphorylation networks. *Bioinformatics* **30,** 141–142 (2014).
11. Perfetto, L. *et al.* SIGNOR: a database of causal relationships between biological entities. *Nucleic Acids Res.* **44,** D548-554 (2016).
12. Garcia-Alonso, L. *et al.* Transcription Factor Activities Enhance Markers of Drug Sensitivity in Cancer. *Cancer Res.* **78,** 769–780 (2018).
13. Subramanian, A. *et al.* Gene set enrichment analysis: a knowledge-based approach for interpreting genome-wide expression profiles. *Proc. Natl. Acad. Sci. U.S.A.* **102,** 15545–15550 (2005).
