# Supplementary figures and images for: Adipocyte-secreted BMP8b mediates adrenergic-induced remodeling of the neuro-vascular network in adipose tissue
Source: Nat Commun. 2018 Nov 26;9:4974. doi: 10.1038/s41467-018-07453-x (PMC6255810; doi:10.1038/s41467-018-07453-x)

Phosphorylation site

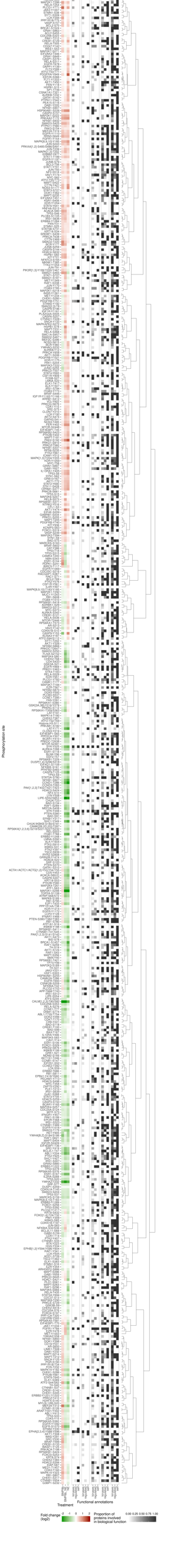

Supplement: Supplementary file 2 — Supplementary Data 1 [file 41467_2018_7453_MOESM2_ESM.pdf]

## Treatments

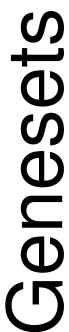

Supplement: Supplementary file 3 — Supplementary Data 2 [file 41467_2018_7453_MOESM3_ESM.pdf]

Phosphorylation site

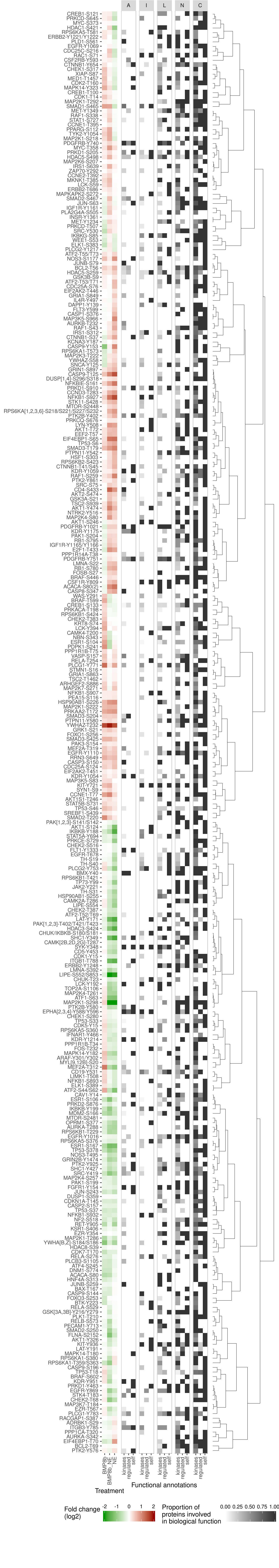

Supplement: Supplementary file 4 — Supplementary Data 3 [file 41467_2018_7453_MOESM4_ESM.pdf]

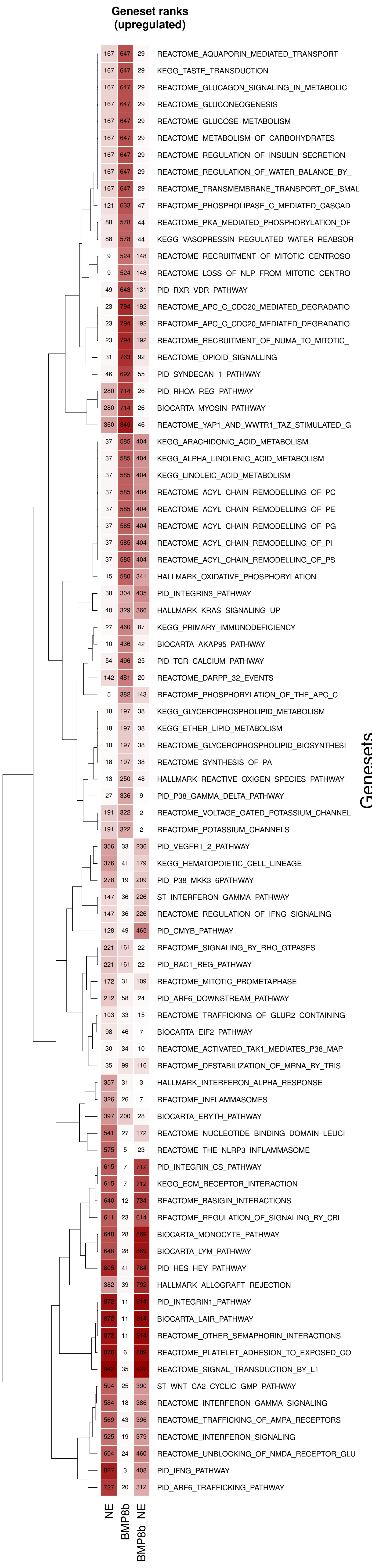

Supplement: Supplementary file 5 — Supplementary Data 4 [file 41467_2018_7453_MOESM5_ESM.pdf]
